# Supplementary material for: Modular detergents tailor the purification and structural analysis of membrane proteins including G-protein coupled receptors
Source: Nat Commun. 2020 Jan 28;11:564. doi: 10.1038/s41467-020-14424-8 (PMC6987200; doi:10.1038/s41467-020-14424-8)
Supplement: Supplementary file 1 — Supplementary Information [file 41467_2020_14424_MOESM1_ESM.pdf]

Supplementary Information

Modular Detergents Tailor the Purification and Structural Analysis of Membrane Proteins Including G-protein Coupled Receptors

Urner *et al*.

1. Supplementary Figures

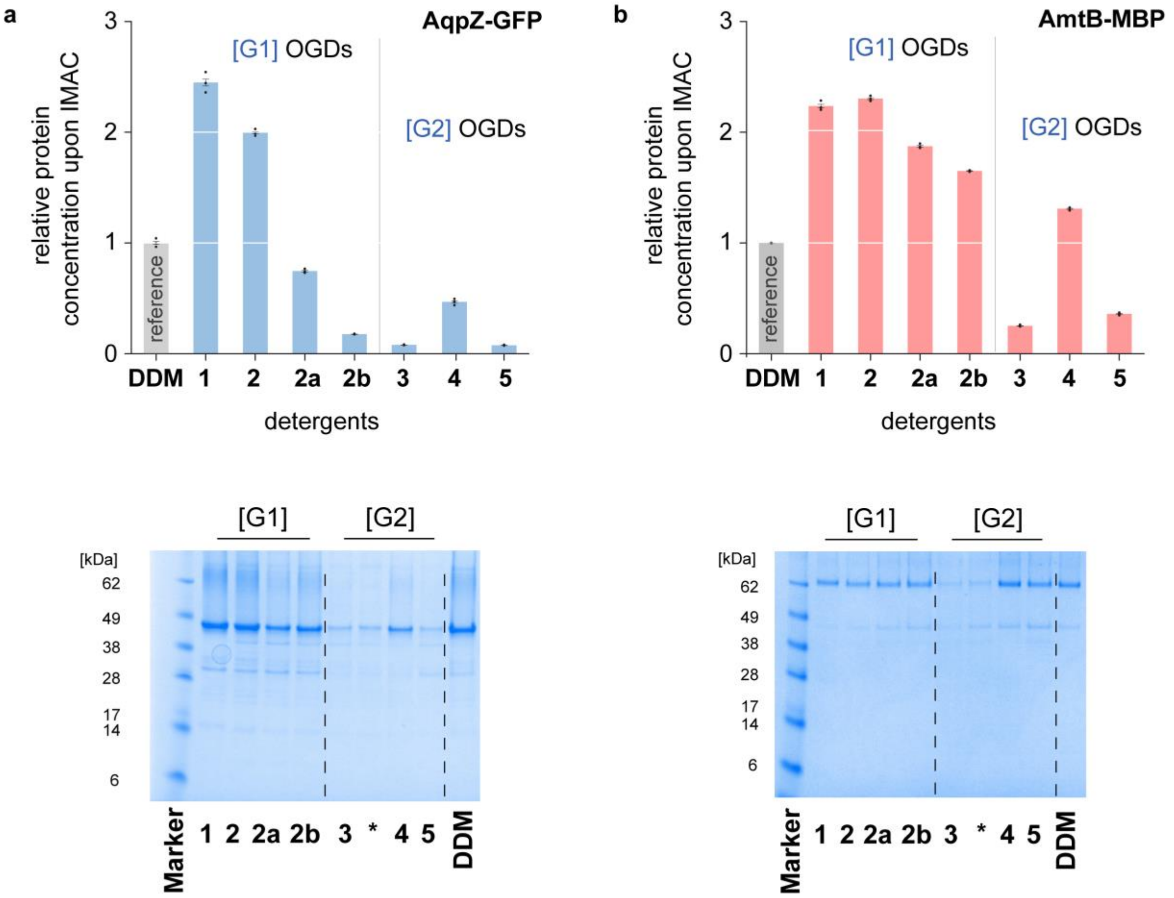

Supplementary Figure 1. Purification of AqpZ-GFP and AmtB-MBP with different detergents. Higher relative protein concentrations were obtained upon IMAC purification of **a** AqpZ-GFP and **b** AmtB-MBP with the [G1] OGD regioisomer mixtures **1** and **2**. The SDS page images shown below confirm that isolated protein-detergent mixtures were of comparable purity. The gel positions labeled with asterisks refer to samples that were purified by [G2]-triazole-C18. This detergent batch serves as control and indicates that changing the linker between head and tail does not improve protein purification when [G2] OGD regioisomer mixtures with linear tails are used. Relative protein concentrations were plotted with standard deviation (± s.d., n = 3). Source data are provided as a Source Data file.

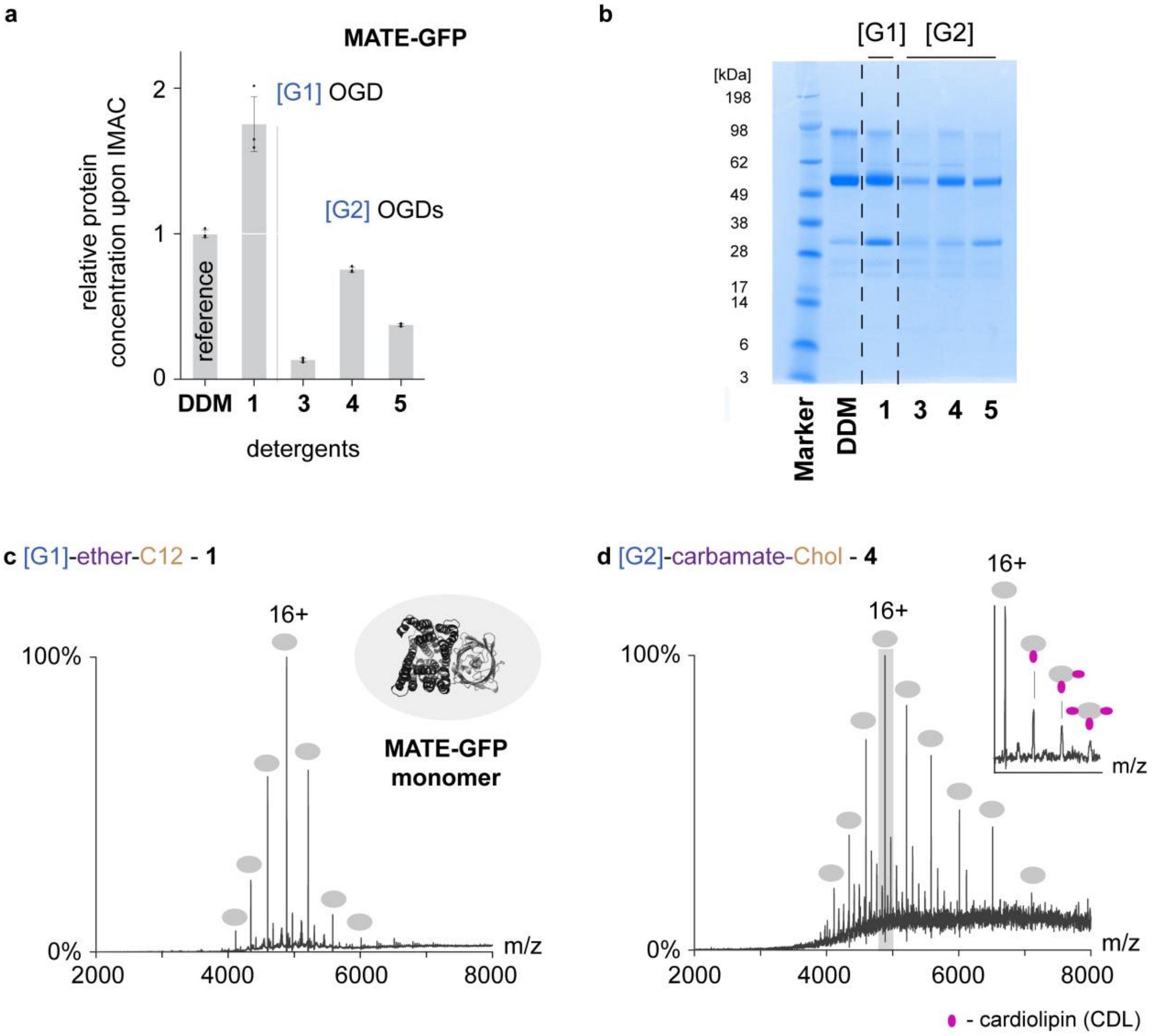

Supplementary Figure 2. Purification and native MS analysis of MATE-GFP. a Higher relative protein concentrations were obtained from [G1] OGD regioisomer mixture **1**. Among the [G2] OGD regioisomer mixtures, higher relative protein concentrations were obtained for lipid-like tails, such as in the cases of **4** and **5**. **b** SDS page analysis confirms that isolated protein-detergent mixtures were of comparable purity. **c** Subsequent native MS analysis revealed reduced intensities of protein-lipid complexes when MATE-GFP was purified with [G1] OGD regioisomer mixture **1**. Substitution of the head group and tail in **d** facilitates the detection of MATE-GFP in complex with membrane lipids. Spectra were acquired using similar instrument conditions (HCD voltage: 200 V). MATE-GFP monomers are indicated by grey circles and cardiolipin by violet circles. Relative protein concentrations were plotted with standard deviation (± s.d., *n* = 3). Source data are provided as a Source Data file.

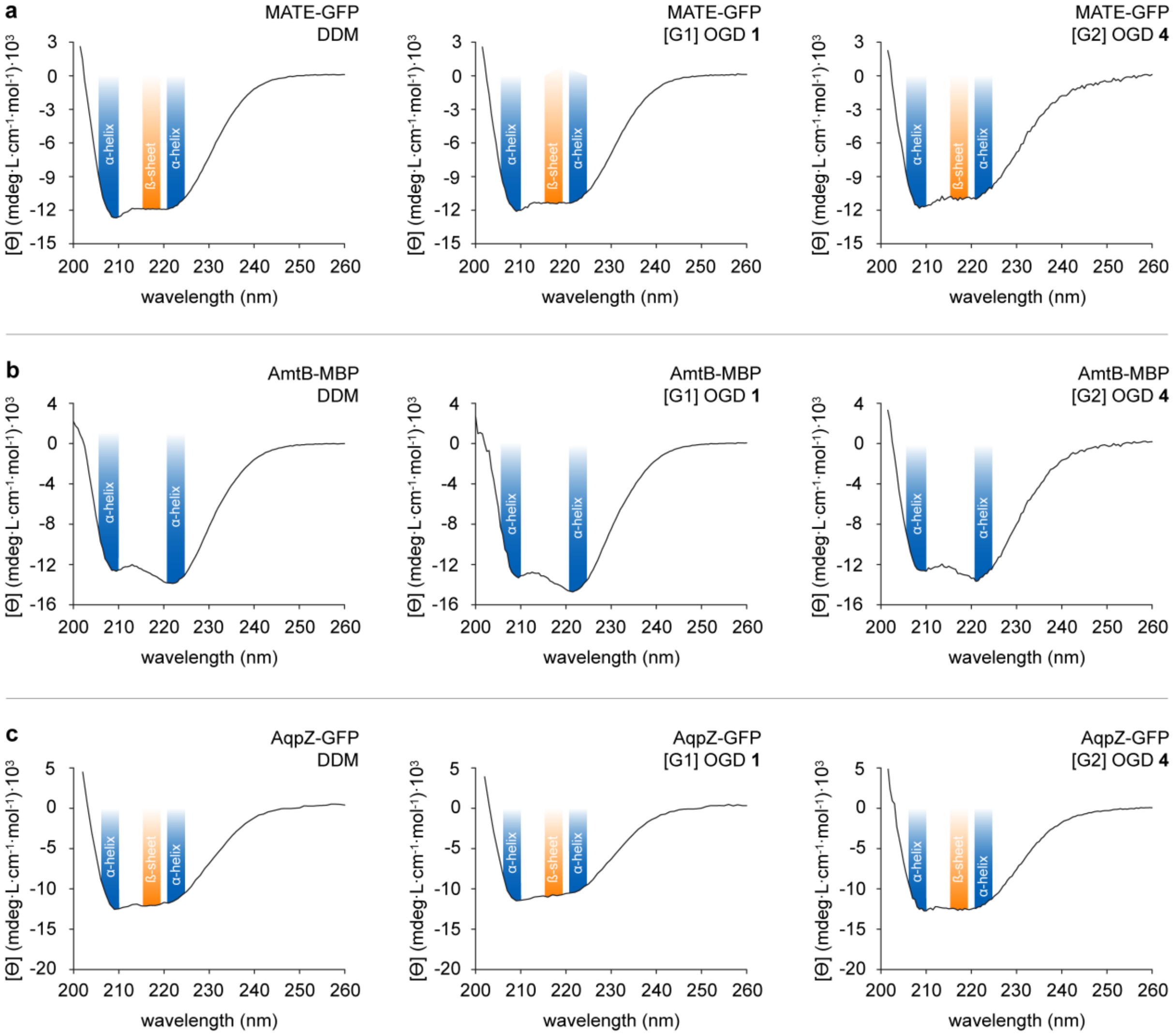

Supplementary Figure 3. Investigating secondary structures of membrane proteins by CD spectroscopy. The CD spectra of **a** MATE-GFP and **c** AqpZ-GFP obtained upon purification with DDM as well as the [G1] and [G2] OGD regioisomer mixtures **1** and **4** show broad CD signals, which display an average of alpha-helical and beta-sheet secondary structures. MATE and AqpZ contain mainly alpha-helical secondary structures, while GFP tags contain mainly beta-sheet secondary structures. The CD spectra obtained in **b** AmtB-MBP are characteristic for alpha-helical secondary structures, which is the main secondary structure of this protein construct. The mean residue ellipticy (ϴ) minima expected for alpha-helical structures (208 nm, 222 nm) and beta-sheet structures (217 nm) are labeled with blue and orange bands, respectively. OGDs preserve native secondary structural elements of membrane proteins during extraction and IMAC purification. Source data are provided as a Source Data file.

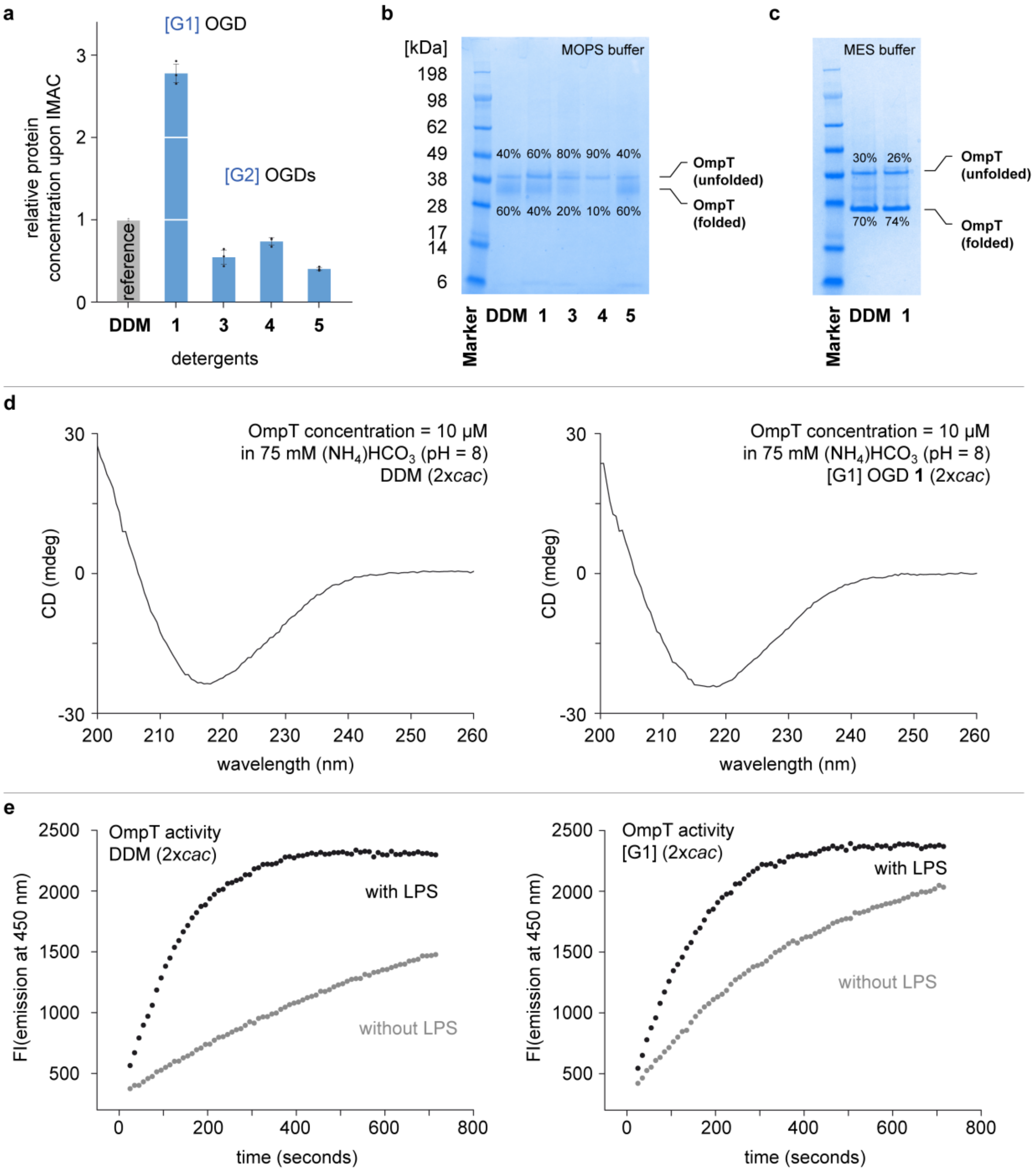

Supplementary Figure 4. Refolding and functional analysis of OmpT. The G236K/K237G mutant of the outer membrane protein T (OmpT) was overexpressed in *E. coli* and purified from inclusion bodies[1] with DDM and OGD batches **1**, **3**, **4**, and **5**. **a** Higher relative protein quantities were obtained upon purification with [G1] regioisomer mixture **1**. **b** SDS page analysis confirmed that isolated protein-detergent mixtures were of comparable purity and different ratios between folded and unfolded OmpT were obtained. Higher relative proportions of folded OmpT were obtained in the case of DDM. However, relative protein quantities obtained from the [G1] OGD regioisomer mixture **1** were about three times higher, thus leading to higher absolute amounts of refolded OmpT under comparable purification conditions. **c** The amount of folded OmpF in samples purified with DDM and **1** could be enriched to approximately 70% using IMAC. **d** CD spectroscopy analysis of the samples shown in **c** revealed a similar high amount of beta-sheet secondary structures. **e** Time-dependent fluorescence analysis revealed a similar proteolytic activity of both samples in the presence of smooth lipopolysaccharide (S-LPS) and the self-quenching fluorescent peptide Abz-ARRAY-Tyr(NO_2)-NH_2 (see Methods). Activity was reduced in the absence of S-LPS, thus underlining that the LPS-mediated protease mechanism is acting in both detergent environments[1]. Samples were not boiled before SDS page analysis. Relative intensities determined from SDS page band intensities between unfolded and folded OmpT are given in percentage (%). Relative protein concentrations were plotted with standard deviation (± s.d., $n = 3$). Source data are provided as a Source Data file.

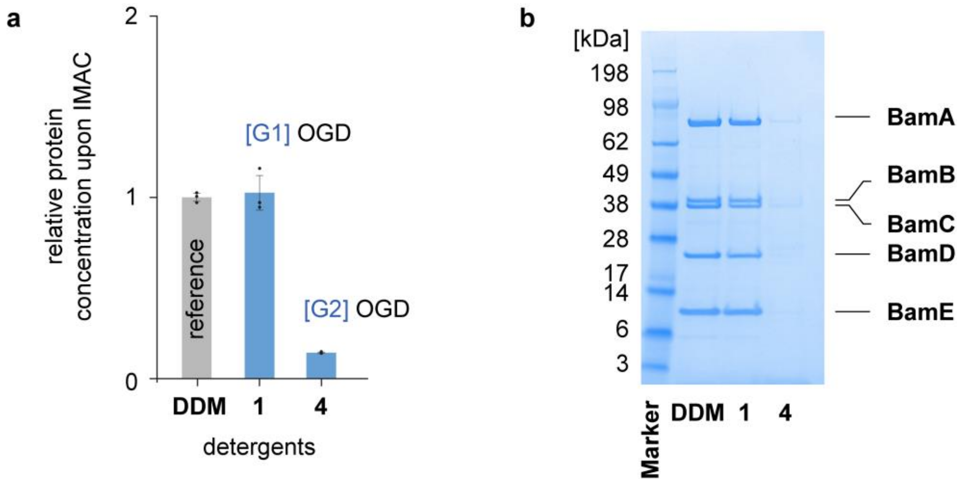

Supplementary Figure 5. Purification of the beta-barrel assembly machinery (BAM) complex. a Higher relative protein concentrations were obtained upon purification with DDM and [G1] OGD regioisomer mixture **1**. **b** SDS page analysis confirmed that all five subunits were co-purified during IMAC, including BamA, BamB, BamC, BamD, and BamE. Only BamE was overexpressed with a polyhistidine tag. Therefore, the SDS page data underline that subunit interactions have been preserved during extraction and IMAC purification. Relative protein concentrations were plotted with standard deviation (± s.d., $n = 3$). Source data are provided as a Source Data file.

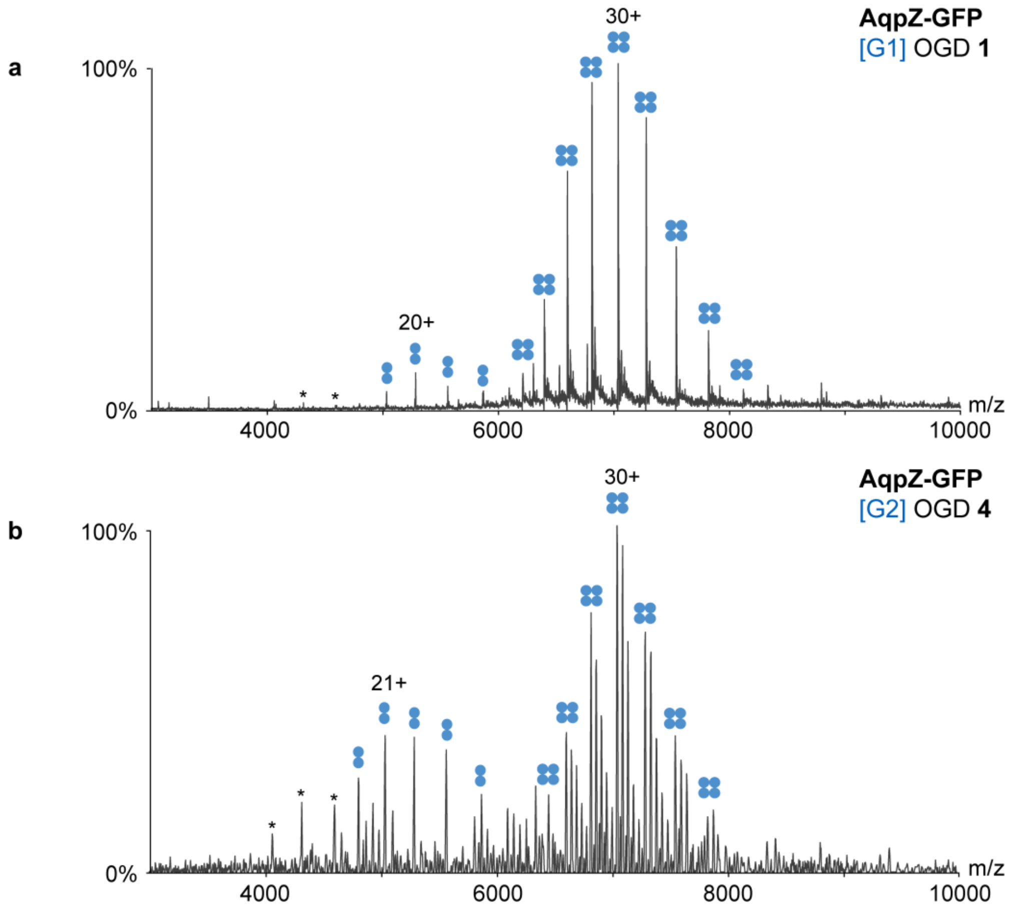

Supplementary Figure 6. Native MS analysis of AqpZ-GFP upon isolation from cell membranes. Mass spectra of AqpZ-GFP were obtained upon extraction and IMAC purification with **a** [G1] OGD regioisomer mixture **1** and **b** [G2] OGD regioisomer mixture **4**. In addition to the native tetrameric state, also dimers are obtained. Impurities are labeled with an asterisk. The relative MS intensities of dimer and tetramer are 5:95 in the case of [G1] OGD regioisomer mixtures **1** and 15:85 in case of [G2] OGD regioisomer mixture **4**. Spectra were acquired using similar instrument conditions (HCD voltage: 200 V). AqpZ-GFP dimers are indicated by two blue circles and tetramers by four blue circles.

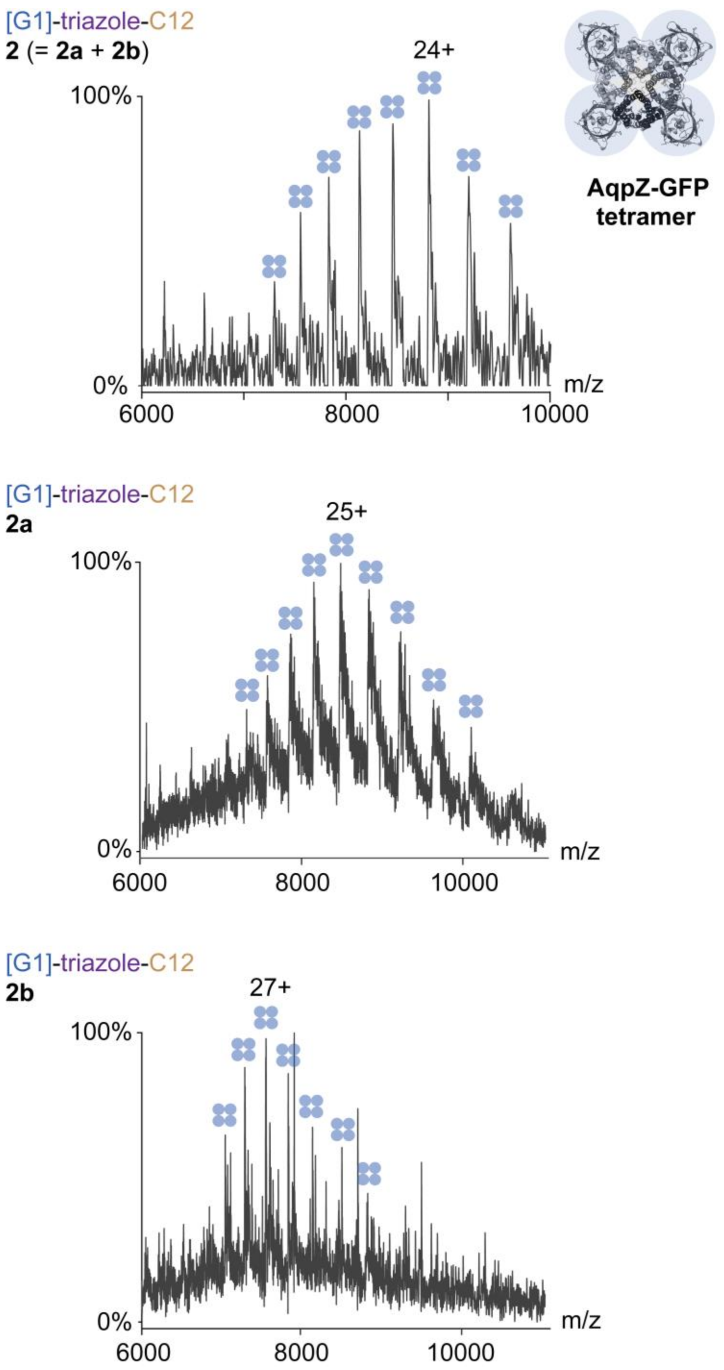

Supplementary Figure 7. Native MS analysis of AqpZ-GFP upon isolation from cell membranes. Mass spectra were obtained after extraction and IMAC purification with [G1] OGD batches **2**, **2a**, and **2b**. Mass spectra were acquired using similar instrument conditions (HCD voltage: 200 V). AqpZ-GFP tetramers are indicated by four blue circles.

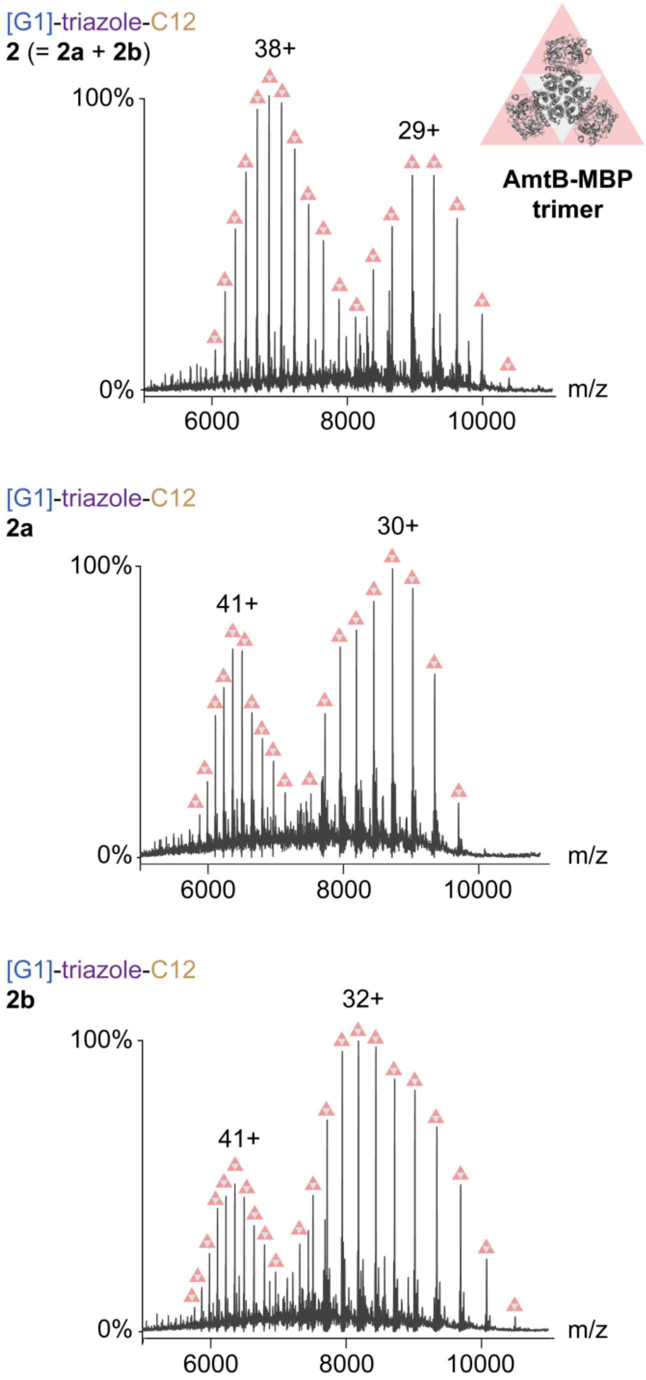

Supplementary Figure 8. Native MS analysis of AmtB-MBP upon isolation from cell membranes. Mass spectra were obtained upon purification with [G1] OGD batches **2**, **2a**, and **2b**. Mass spectra were acquired using similar instrument conditions (HCD voltage: 200 V). Bimodal charge state distributions are likely a consequence of charge-stripping effects or partial unfolding of the MBP tags. For further information about charge-stripping effects see Reading *et al.*[2] AmtB-MBP trimers are indicated by pink triforces.

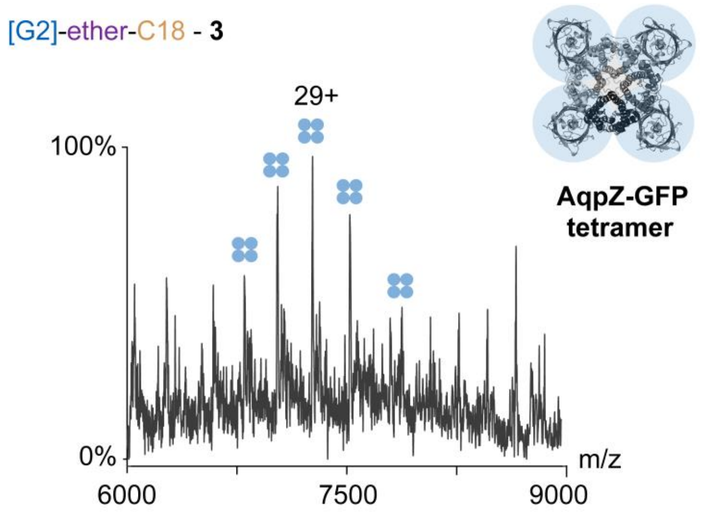

Supplementary Figure 9. Native MS analysis of AqpZ-GFP upon isolation from cell membranes. The mass spectrum of tetrameric AqpZ-GFP was obtained upon extraction and IMAC purification with the [G2] OGD regioisomer mixture **3**. Spectra were acquired using similar instrument conditions (HCD voltage: 200 V). AqpZ-GFP tetramers are indicated by four blue circles.

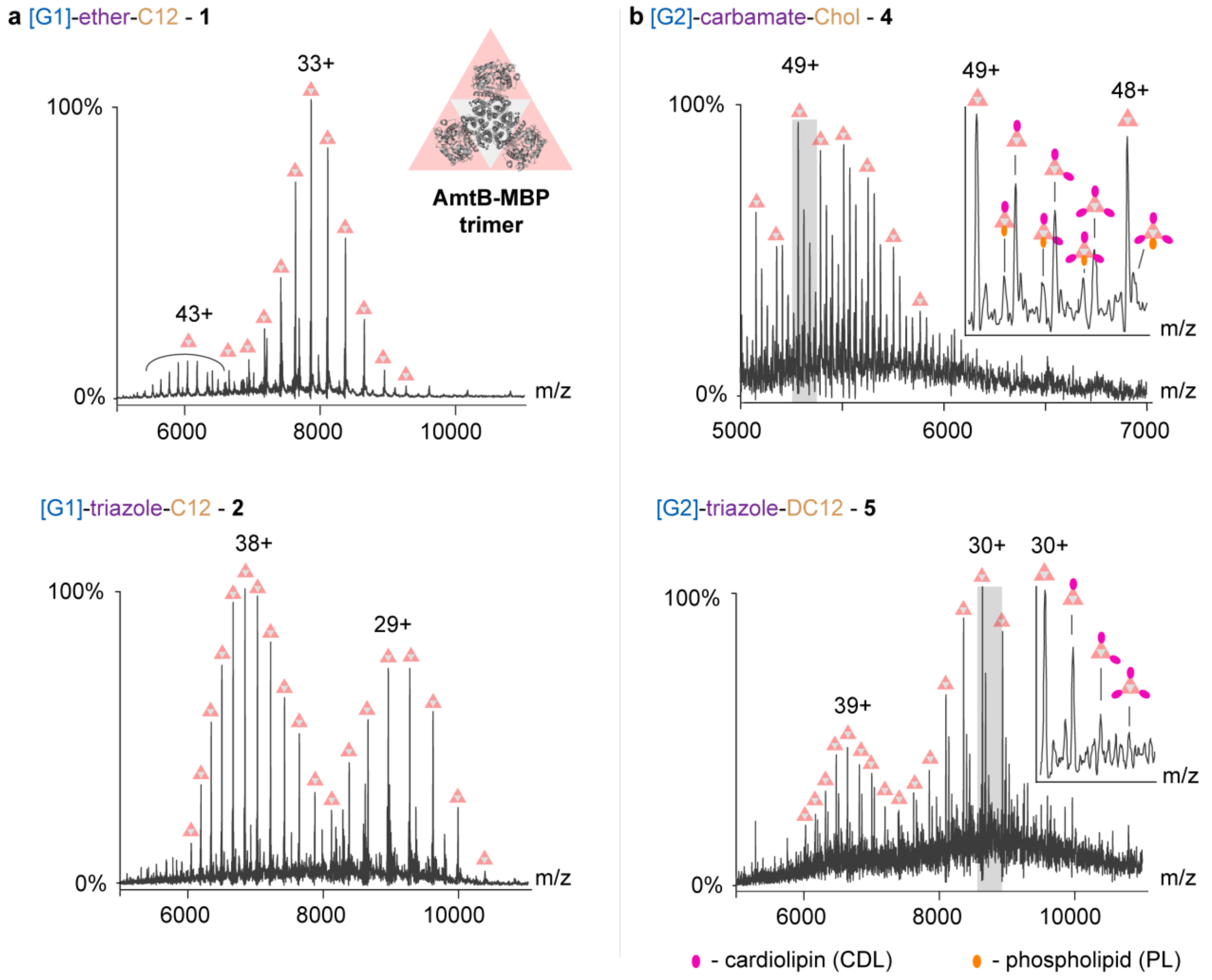

Supplementary Figure 10. Native MS analysis of AmtB-MBP upon isolation from cell membranes. **a** [G1] OGD regioisomer mixtures facilitate protein extraction and lead to charge reduction when the ether linker (top) is substituted for triazol (bottom). In both cases, no protein lipid-complexes are observed. Substitution of the [G2] head group and hydrophobic tail in **b** enables the detection of the protein in complex with membrane lipids. Increasing the basicity of the linker leads to charge reduction, irrespective of head group and tail. Bimodal charge state distributions are likely a consequence of charge-stripping effects or partial unfolding of the MBP tags. For further information about charge-stripping effects see Reading *et al.*[2] Spectra were acquired using similar instrument conditions (HCD voltage: 200 V). AmtB-MBP trimers are indicated by pink triforces, cardiolipins by violet circles, and phospholipids by orange circles.

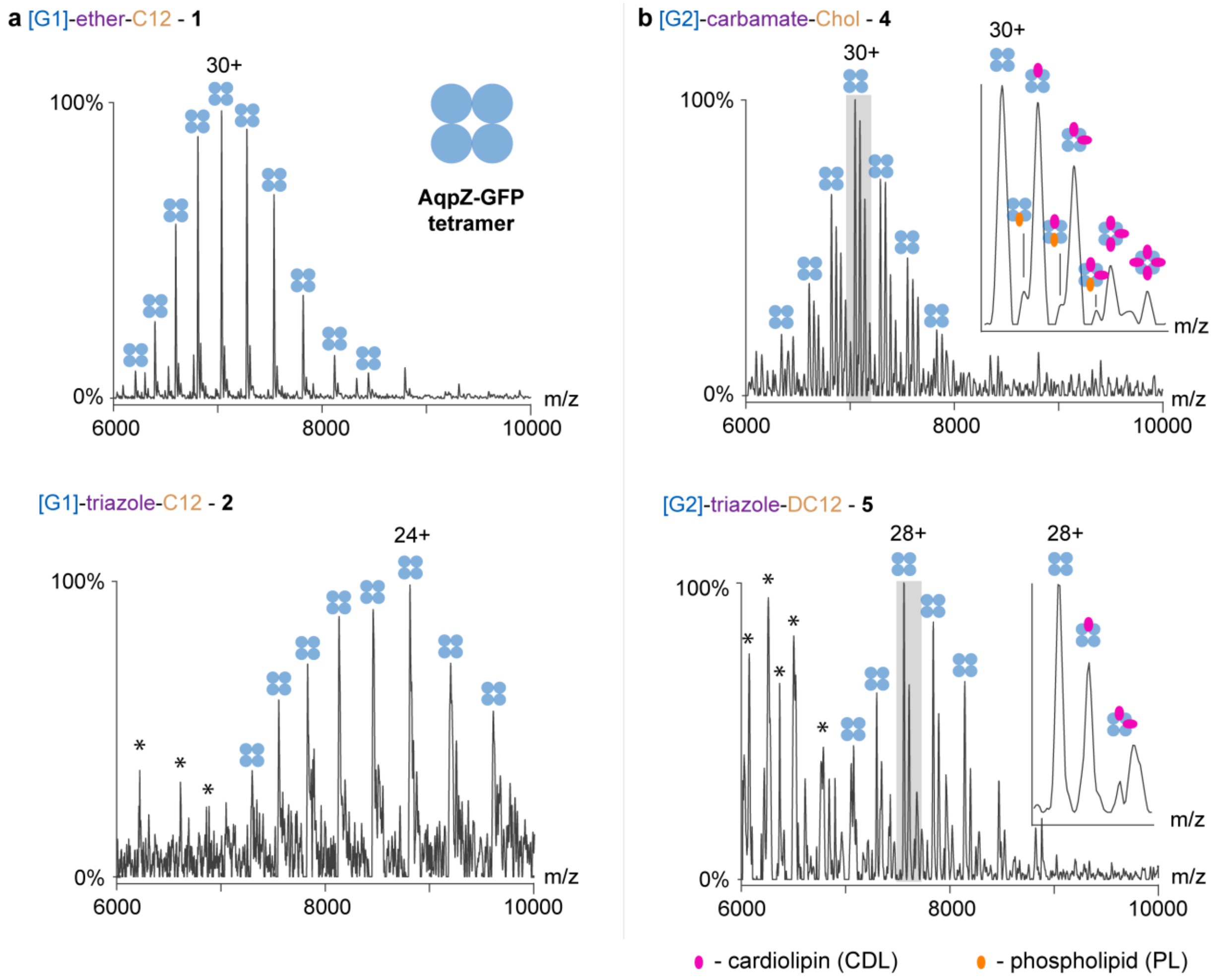

Supplementary Figure 11. Native MS analysis of AqpZ-GFP upon isolation from cell membranes. **a** [G1] OGD regioisomer mixtures facilitate protein extraction and lead to charge reduction when the ether linker (top) is substituted for triazol (bottom). In both cases, no protein lipid-complexes are observed. Substitution of the head group and hydrophobic tail in **b** [G2] OGD regioisomer mixtures enabled detection of the protein in complex with structurally relevant membrane lipids. Increasing the basicity of the linker leads to charge reduction, irrespective of head group and tail. Spectra were acquired using similar instrument conditions (HCD voltage: 200 V). Impurities are labeled with an asterisk. AqpZ-GFP tetramers are indicated by four blue circles, cardiolipins by violet circles, and phospholipids by orange circles.

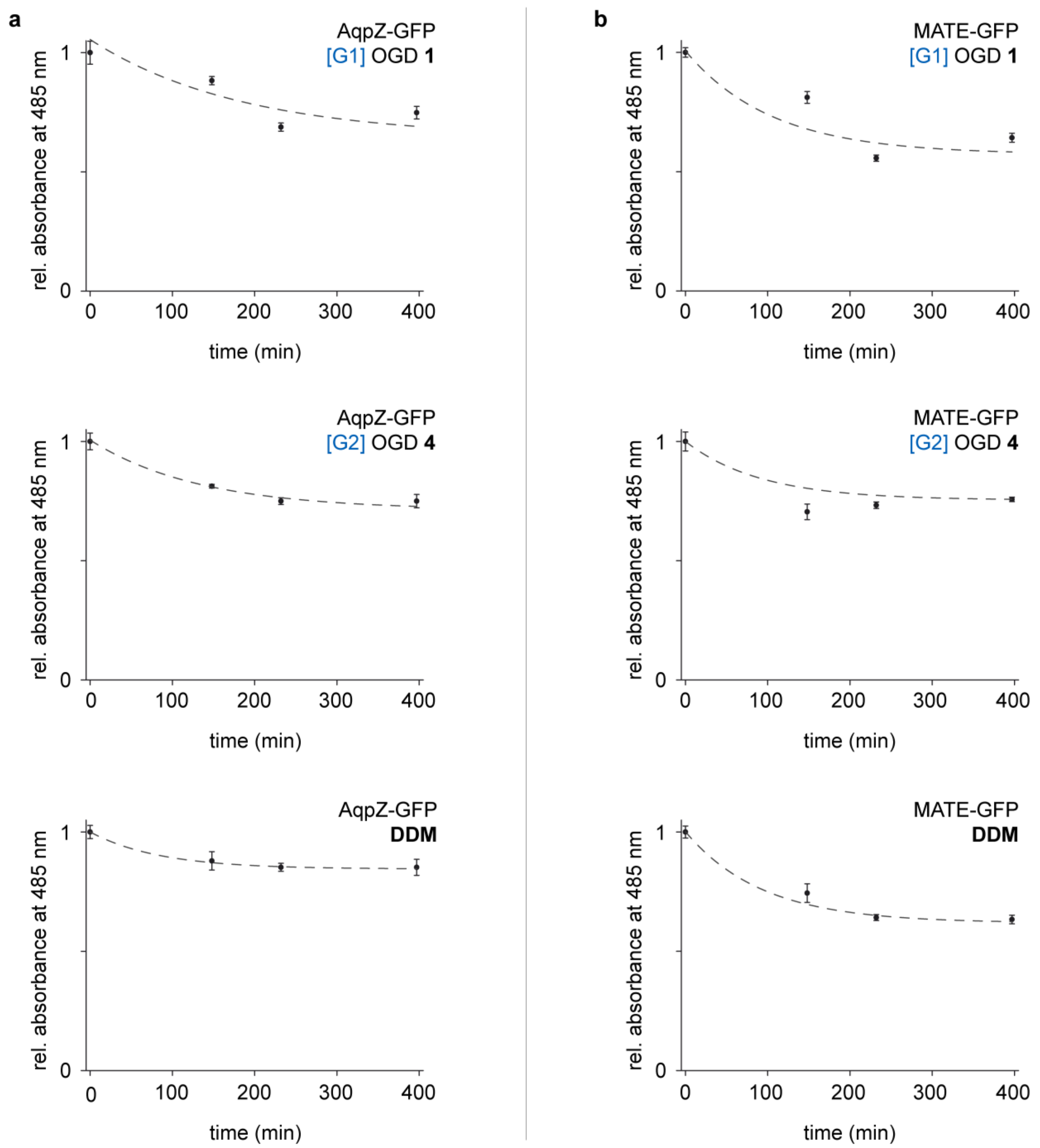

Supplementary Figure 12. Investigating protein stabilities in MS buffer with different detergents. The stability data for **a** MATE-GFP and **b** AqpZ-GFP in MS buffer containing the [G1] and [G2] OGD regioisomer mixtures **1** and **4**, or DDM are shown. Both proteins were purified with **1**, **4**, or DDM and then buffer exchanged into detergent-containing MS buffer. Subsequently, the protein solutions were stored at 4 °C and the protein concentrations were monitored using a microvolume spectrophotometer (DeNovix, United Kingdom). Precipitated protein was spun down by centrifugation. The supernatant absorbance at 485 nm (A_{485}) was determined at different time points after the final buffer exchange (t = 0 min, 148 min, 232 min, 397 min). The A_{485} values were normalized to the initial A_{485} value (t = 0 min) and plotted against the storage time. To better visualize the time-dependent drop of the protein concentration, the data were fitted to exponential functions (dashed lines). The stabilities of AqpZ-GFP and MATE-GFP against precipitation in MS buffer containing **1**, **4**, or DDM were similar under the experimental conditions employed. Relative absorbance values were plotted with standard deviation (± s.d., *n* = 3). Source data are provided as a Source Data file.

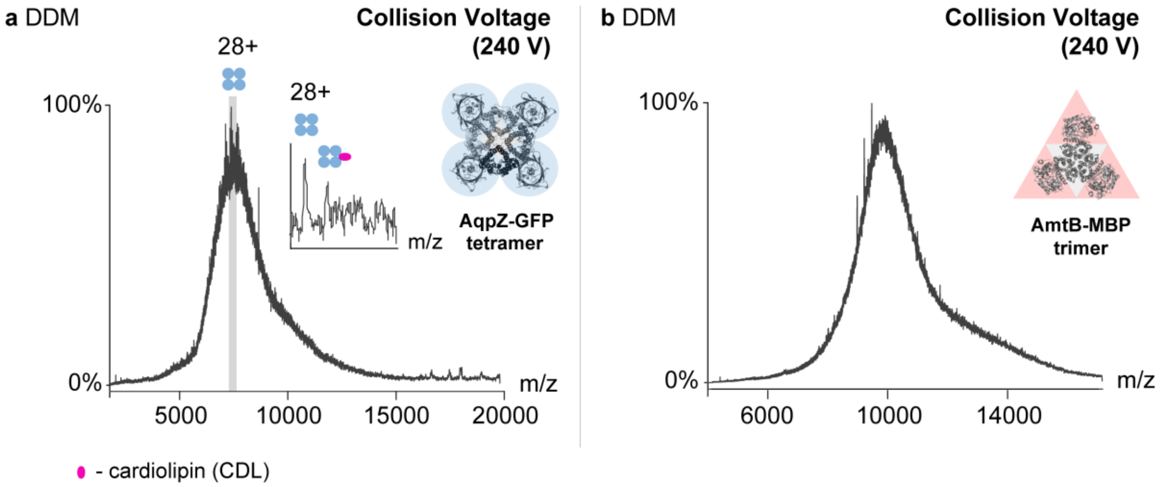

Supplementary Figure 13. Mass spectra of AqpZ-GFP and AmtB-MBP upon purification with DDM. Mass spectra were obtained after extraction and IMAC purification of **a** AqpZ-GFP and **b** AmtB-MBP with DDM. Insufficient detergent removal and poorly resolved spectra are obtained even when higher activation conditions were applied (HCD voltage of 240 V). The zoom shown in **a** shows the partially resolved charge state 28+. AqpZ-GFP tetramers are indicated by four blue circles and cardiolipins by violet circles.

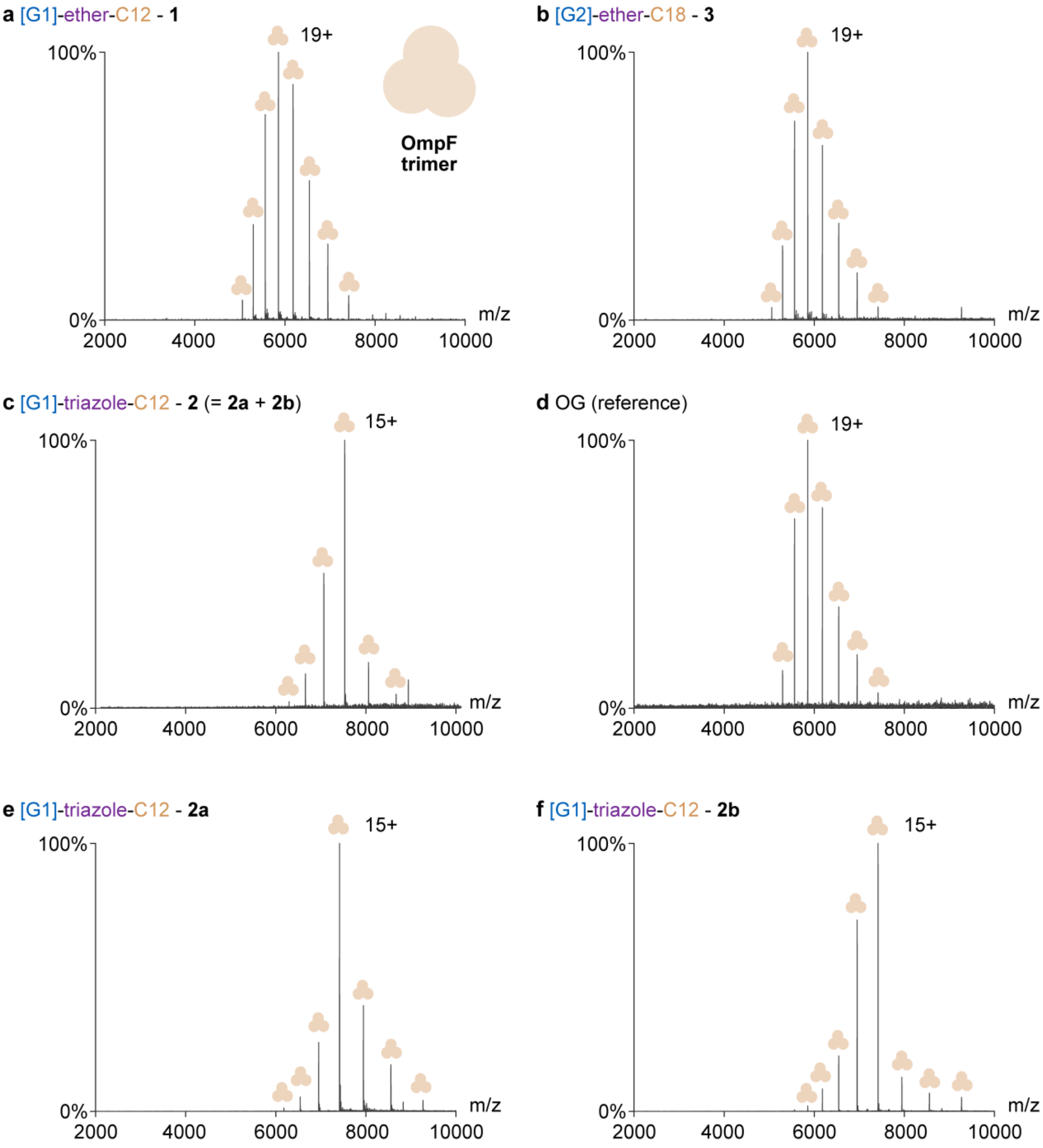

Supplementary Figure 14. Native MS analysis of OmpF. Mass spectra of OmpF were obtained upon exchange from *n*-octyl-β-D-glucopyranoside (OG) to **1**, **2**, **2a**, **2b**, **3** and OG. **a** – **b** Varying the size of the dendritic head group and hydrophobic tail between **1** and **3** did not alter the charge states of OmpF. **d** Similar to the reference detergent OG, these detergents are not associated with protein charge reduction. **a**, **c** Only a substitution of the ether moiety in **1** by the more basic triazole in **2** leads to a substantial charge reduction of OmpF. **e** – **f** Similar charge reduction is obtained for the individual regioisomers **2a** and **2b**, which confirms that protein charge reduction is independent from the structure of the triglycerol head group. Spectra were acquired using similar instrument conditions (HCD voltage: 200 V).

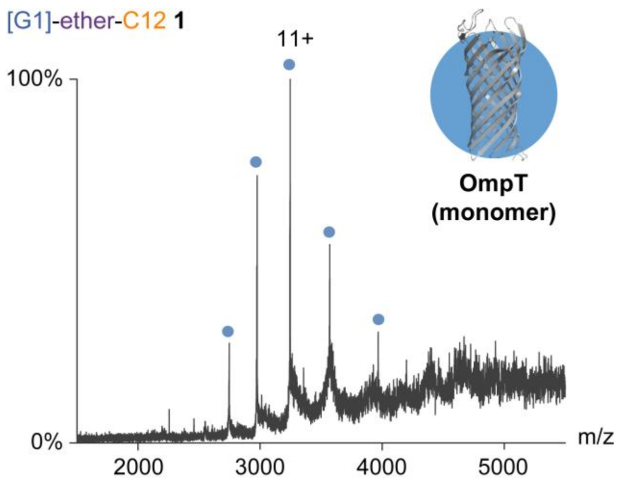

Supplementary Figure 15. Mass spectrum of OmpT (G236K/K237G). The mass spectrum was obtained upon refolding with the [G1] OGD regioisomer mixture **1**.

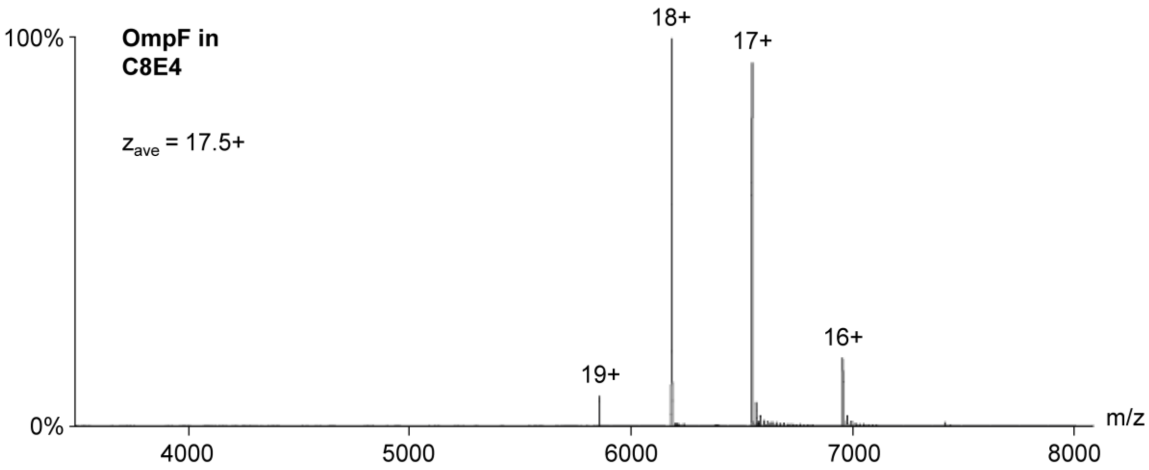

Supplementary Figure 16. Mass spectrum of OmpF. The mass spectrum was obtained after detergent exchange from OG to C8E4.

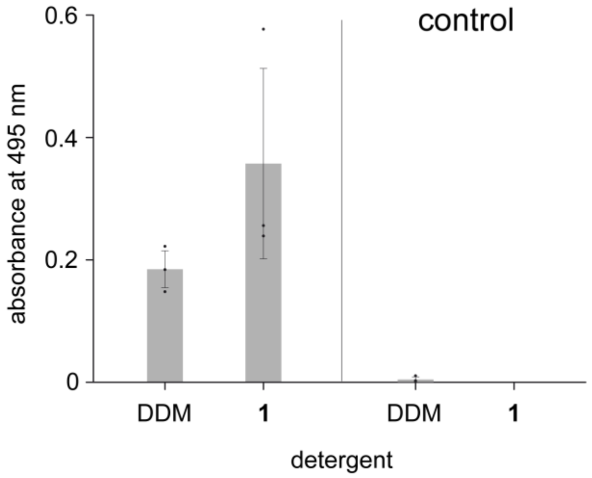

Supplementary Figure 17. Investigating binding between NTSR1 and [5,6-FAM-NT(8-13)]. NTSR1 (7 µM) was solubilized in binding buffer (0.01 M Hepes, 0.15 M NaCl, 10 *v/v* glycerol, pH = 8) containing either the [G1] OGD regioisomer mixture **1** or DDM. [5,6-FAM-NT(8-13)] (2.5 eq) was added and the mixture was incubated on ice for 15 minutes. The excess of agonist was removed with a Micro Bio-Spin column (MWCO = 7 kDa, Bio-Rad). Remaining sample absorbance (A_{495}) was determined by UV/VIS spectroscopy and revealed similar A_{495} values for **1** and DDM. Control experiments with protein-free samples revealed A_{495} values close to zero and are labeled with an asterisk. All experiments were done in triplicate and results were plotted with standard deviation (± s.d., $n = 3$). In case of OGD regioisomer mixture **1** the error bars are relatively large due to an outliner (absorbance at 495 nm ~ 0.6, see overlaid dot blot). It is possible that this sample became less diluted than the others due to variations in Bio-Spin column handling or batch to batch variation of column properties. Source data are provided as a Source Data file.

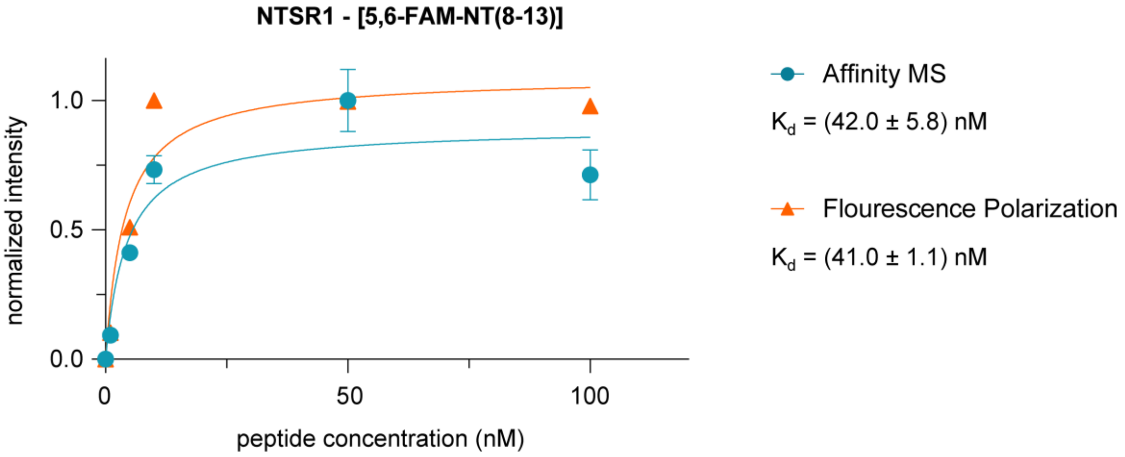

Supplementary Figure 18. Investigating the K_d value of NTSR1 and [5,6-FAM-NT(8-13)]. Affinity MS and fluorescence polarization revealed K_d values in the nanomolar range when the receptor was purified with the [G1] OGD regioisomer mixture **1**. Normalized intensities obtained from affinity MS measurements were plotted with standard deviation (± s.d., $n = 3$). Source data are provided as a Source Data file.

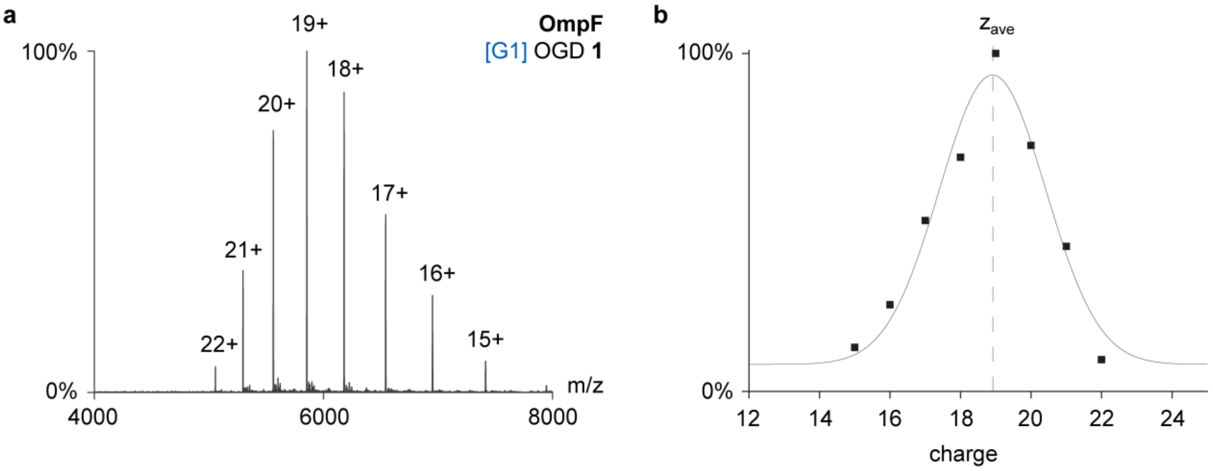

Supplementary Figure 19. Calculating the average charge states (z_{ave} values) of membrane proteins. The z_{ave} values were calculated as described exemplarily here[3]: The mass spectrum shown in **a** was obtained from OmpF upon detergent exchange into the [G1] OGD regioisomer mixture **1**. To calculate z_{ave}, the spectral intensities of the protein charge states shown in **a** were extracted and plotted against the protein charge states (apo form), such as shown in **b**. The data were fitted to a Gaussian function and the charge state (x-value) at maximum intensity was takes as the z_{ave} value.

2. Supplementary Tables

Supplementary Table 1. Evaluating the significance of AmtB-MBP extraction results. P-tests were performed to validate the statistical significance of differences in relative protein concentrations obtained from AmtB-MBP upon extraction and IMAC with [G1] OGD regioisomer mixture **2** (= **2a** + **2b**) and [G1] OGD regioisomers **2a** and **2b**. Differences in relative protein concentrations are considered as significant for P-values lower than 0.05. Based on the Analysis of Variance (ANOVA) test, all data set combinations fulfill this criterion. We conclude that differences in relative protein concentrations between **2**, **2a**, and **2b** are significant, although they are less pronounced than in case of AqpZ. Source data are provided as a Source Data file.

OGD Batch Comparison	P-value
**2** and **2a**	0.002
**2** and **2b**	0.0005
**2a** and **2b**	0.004

Supplementary Table 2. Summary of native MS data. Protein extraction and MS data obtained for AqpZ-GFP, AmtB-MBP, MATE-GFP, OmpT, and MBP-NTSR1-TrxA upon extraction from *E. coli* membranes, purification, and nESI-MS analysis. The following data are summarized: protein construct, structural abbreviation of the detergent, protein concentration in solution upon IMAC relative to DDM [P], average charge state (z_{ave}), lipid masses (± s.d., $n = 6$) calculated from MS data, and assigned lipids according to Laganowsky *et al.*[4] Detergent masses are given for comparison. Source data are provided as a Source Data file.

**Protein**	**Detergent**	**[P]**	**z_{ave}**	**Lipid Mass [Da]**	**Lipid**	**Detergent Mass [Da]**
AqpZ-GFP	DDM	1	(28+)*	(1427 ± 6)	CDL	510.3
	**1** [G1]-ether-C12	2.4	30+	-	-	-
	**2** [G1]-triazole-C12	2.0	25+	-	-	-
	**2a** [G1]-triazole-C12	0.7	25+	-	-	-
	**2b** [G1]-triazole-C12	0.2	27+	-	-	-
	**3** [G2]-ether-C18	0.1	29+	-	-	-
	**4** [G2]-carbamate-Chol	0.5	30+	(901 ± 3), (1414 ± 3)	PL, CDL	947.6
	**5** [G2]-triazole-DC12	0.1	28+	(1372 ± 4)	CDL	1055.6
AmtB-MBP	DDM	1	-	-	-	-
	**1** [G1]-ether-C12	2.2	43+/32+	-	-	-
	**2** [G1]-triazole-C12	2.3	38+/29+	-	-	-
	**2a** [G1]-triazole-C12	1.9	38+/27+	-	-	-
	**2b** [G1]-triazole-C12	1.7	38+/27+	-	-	-
	**3** [G2]-ether-C18	0.2	-	-	-	-
	**4** [G2]-carbamate-Chol	1.3	48+	(983 ± 3), (1400 ± 1)	PL, CDL	947.6
	**5** [G2]-triazole-DC12	0.4	40+/30+	(1394 ± 1)	CDL	1055.6
MATE-GFP	DDM	1	-	-	-	-
	**1** [G1]-ether-C12	1.8	16+	(720 ± 14)	PL	408.3
	**3** [G2]-ether-C18	0.1	*n.t.*	-	-	-
	**4** [G2]-carbamate-Chol	0.8	16+	(1386 ± 7)	CDL	947.6
	**5** [G2]-triazole-DC12	0.4	*n.t.*	-	-	-
OmpT	DDM	1	(10+)[5]**	-	-	-
	**1** [G1]-ether-C12	2.8	11+	-	-	-
	**3** [G2]-ether-C18	0.5	*n.t.*	-	-	-
	**4** [G2]-carbamate-Chol	0.7	*n.t.*	-	-	-
	**5** [G2]-triazole-DC12	0.4	*n.t.*	-	-	-
MBP-NTSR1-TrxA	DDM	1	-	-	-	-
	**1** [G1]-ether-C12	1.3	20+	(744 ± 24)	PL	408.3
	**4** [G2]-carbamate-Chol	0.7	20+	(728 ± 26)	PL	947.6

* - This value displays the most abundant charge state obtained from a partially resolved spectrum upon purification with DDM (see Supplementary Fig. 11).

n.t. **–** MS analysis upon solubilization and purification was not tested.

** - This value displays the most abundant charge state obtained upon solubilization with DDM (see literature[5]).

Supplementary Table 3. Critical aggregation concentration (cac) values of OGD batches. The cac values of the OGD batches **1** – **5** were determined by DLS. The regioisomer ratios result from the starting material that is used for the synthesis of OGDs. Regioisomer ratios determined for final products and intermediates are given in the synthetic procedures. Further information about the analysis of regioisomer ratios is given in the sub-chapter Quantification of Regioisomer Proportions. Source data are provided as a Source Data file.

**[G1] OGD mixture**	**cac (mM)**	**[G2] OGD mixture**	**cac (mM)**
[G1]-ether-C12 (**a**:**b**, 6:4) **1**	0.70	[G2]-ether-C18 (**aa**:**ab**:**bb**, 4:4:1) **3**	0.30
[G1]-triazole-C12 (**a**:**b**, 6:4) **2**	0.55	[G2]-carbamate-Chol (**aa**:**ab**:**bb**, 9:4:1) **4**	0.16
[G1]-triazole-C12 **2a**	0.47	[G2]-triazole-DC12 (**aa**:**ab**:**bb**, 9:4:1) **5**	0.06
[G1]-triazole-C12 **2b**	0.39		

Supplementary Table 4. Detergent concentrations applied for purification and native MS experiments. Detergent concentrations applied for detergent exchange (OmpF), membrane solubilization, IMAC purification, and MS buffers (AqpZ, AmtB, MATE, OmpF, BAM complex, NTSR1). Similar detergent concentrations were used for the refolding and IMAC purification of OmpT**.** For MS experiments with [G1] OGD **1** the detergent concentration was adjusted to 1xcac, because the cac of this detergent batch is considerably higher than the cac values of the other OGD batches (see Supplementary Table 3). However, further tests confirmed that adjusting the [G1] OGD **1** concentration to 2xcac does not affect the quality of the mass spectra. Source data are provided as a Source Data file.

**Detergent Abbreviation**	**Detergent Exchange OG → 1 – 3**	**Membrane Solubilization**	**IMAC Wash Buffer**	**IMAC Elution Buffer**	**MS Buffer**
**1** [G1]-ether-C12	1xcac	1w% (36xcac)	2xcac	2xcac	1xcac
**2** [G1]-triazole-C12	2xcac	1w% (39xcac)	2xcac	2xcac	2xcac
**2a** [G1]-triazole-C12	2xcac	1w% (44xcac)	2xcac	2xcac	2xcac
**2b** [G1]-triazole-C12	2xcac	1w% (53xcac)	2xcac	2xcac	2xcac
**3** [G2]-ether-C18	2xcac	1w% (44xcac)	2xcac	2xcac	2xcac
**4** [G2]-carbamate-Chol	-	1w% (67xcac)	2xcac	2xcac	2xcac
**5** [G2]-triazole-DC12	-	1w% (167xcac)	2xcac	2xcac	2xcac
DDM	-	1w% (115xcac)	2xcac	2xcac	2xcac

Supplementary Table 5. Summary of protein masses determined by native MS. Overview of protein species detected by MS, theoretical masses according to amino acid sequence, experimental masses determined by MS, and detergent batches. Experimental data are given with standard deviation (± s.d., *n* = 3). Source data are provided as a Source Data file.

Protein	Theoretical Mass [kDa]	Experimental Mass [kDa]	Detergent
AqpZ-GFP	211.052	210.9 ± 0.30	**1** [G1]-ether-C12
(tetramer)		211.3 ± 0.10	**2** [G1]-triazole-C12
		211.4 ± 0.01	**2a** [G1]-triazole-C12
		211.0 ± 0.03	**2b** [G1]-triazole-C12
		211.1 ± 0.10	**3** [G2]-ether-C18
		211.0 ± 0.10	**4** [G2]-carbamate-Chol
		211.3 ± 0.30	**5** [G2]-triazole-DC12
		211.2 ± 0.50	DDM
AqpZ-GFP	105.526	105.6 ± 0.01	**1** [G1]-ether-C12
(dimer)		105.6 ± 0.05	**4** [G2]-carbamate-Chol
AmtB-MBP	258.914	259.1 ± 0.01	**1** [G1]-ether-C12
(trimer)		259.1 ± 0.01	**2** [G1]-triazole-C12
		259.1 ± 0.01	**2a** [G1]-triazole-C12
		259.2 ± 0.01	**2b** [G1]-triazole-C12
		259.1 ± 0.01	**3** [G2]-ether-C18
		258.9 ± 0.01	**4** [G2]-carbamate-Chol
		259.1 ± 0.01	**5** [G2]-triazole-DC12
MATE-GFP	78.067	78.1 ± 0.01	**1** [G1]-ether-C12
(monomer)		78.1 ± 0.01	**4** [G2]-carbamate-Chol
OmpF	111.253	111.2 ± 0.01	**1** [G1]-ether-C12
(trimer)		111.2 ± 0.01	**2** [G1]-triazole-C12
		111.2 ± 0.01	**2a** [G1]-triazole-C12
		111.2 ± 0.01	**2b** [G1]-triazole-C12
		111.2 ± 0.01	**3** [G2]-ether-C18
		111.2 ± 0.01	OG
		111.2 ± 0.01	C8E4
OmpT (monomer)	35.770	35.7 ± 0.02	**1** [G1]-ether-C12
MBP-NTSR1-TrxA	94.630	94.5 ± 0.05	**1** [G1]-ether-C12
(monomer)		94.3 ± 0.10	**4** [G2]-carbamate-Chol

3. Supplementary Methods

General Remarks Related to Synthesis

OGD structures that are discussed throughout this document are abbreviated by the following formula: "D-L-H." The letter "D" represents the head group either in its acetal-protected state [pGX] or unprotected state [GX]. The letter "X" represents the generation of triglycerol dendron. The letter "L" represents the functional group at the focal point and the letter "H" represents the hydrophobic tail. With the exception of [G1]-ether-C12 **1a**[6] and [G2]-triazole-DC12 **5aa**[7], the here presented OGDs and OGD mixtures have not been published before. The synthesis and functionalization of acetal-protected triglycerol dendrons were performed using previously published synthetic strategies[6, 7, 8, 9, 10].

Chemicals were purchased from Sigma-Aldrich (Germany), Acros Organics (Germany), Alfa Aesar (Germany), Fluka (Germany), Fischer Scientific (Deutschland), Merck (Germany), TCI (Germany), and were used as supplied. Ethyl acetate (EtOAc) and (*n*-hexane) were distilled before they were used. Other solvents, such as methanol (MeOH), dimethylformamide (DMF), dichloromethane (DCM), and tetrahydrofuran (THF), were used as supplied. Dry solvents were purchased in bottles sealed with a septum or tapped from a solvent purification system (MS-SPS-800), which were brought from M. Braun (Germany). Deionized water (H_2O) used for synthesis was used from the tap and was provided by a deionization system installed in the Freie Universität's Institute of Chemistry and Biochemistry. Argon and oxygen were purchased from Linde (Germany) and used as supplied. For working under dry and oxygen-free reactions conditions, chemicals and solvents were handled under argon atmosphere. To support dry conditions the glassware was evacuated, heated up to 300 °C using a heat gun, and filled with argon prior usage.

For reaction monitoring and purification procedures normal phase (NP) thin-layer chromatography (TLC) analysis was applied. NP TLC plates (DC-Fertigfolien ALUGRAM® Xtra SIL G/UV254) based on silica (SiO_2) were purchased from Macherey-Nagel (Germany). Silica gel (60 M) for preparative normal phase column chromatography was purchased from Macherey-Nagel. For NP TLC analysis and manual NP column purification mixtures of organic solvents (*v:v*) were prepared. If necessary, MeOH was added in percent per volume to the prepared mixtures (*v*:*v* + *v*%). TLC plates were either analyzed under UV irradiation (254 nm) using a lamp from CAMAG (Germany) or by staining the TLC plates either with cerium reagent (940 mL H_2O, 60 mL H_2SO_4, 25 g molybdic acid, 10 g cerium(IV) sulfate) or a potassium permanganate solution (250 mL H_2O, 2.5 g potassium permanganate). For the staining process, the TLC plates were fully submerged into the staining solution, excess of staining reagent was wiped off with cellulose, and the plate was heated up to 300 °C with a heat gun until staining was completed.

Mass spectra were acquired on an Agilent 6210 ESI-TOF (ESI-ToF) from Agilent Technologies (Santa Clara, CA, USA). The solvent flow rate was adjusted to 4 µL/min and the spray voltage was set to 4 kV. Drying gas flow rate was set to 15 psi (1 bar). All other parameters were adjusted for a maximum abundance of the relative $[M+H]^+$. The instrument was operated by the Core Facility BioSupraMol of the Freie Universität Berlin. ^{1}H NMR, ^{13}C NMR, and DEPT135 spectra were acquired using the following NMR instruments: Bruker DPX400 (^{1}H NMR: 400 MHz, ^{13}C NMR: 101 MHz), Jeol ECX400 (^{1}H NMR: 400 MHz, ^{13}C NMR: 101 MHz), Jeol ECP 500 (^{1}H NMR: 500 MHz, ^{13}C NMR: 126 MHz), Bruker AVANCEIII500 (^{1}H NMR: 500 MHz, ^{13}C NMR: 126 MHz) or Bruker AVANCEIII700 (^{1}H NMR: 700 MHz, ^{13}C NMR: 175 MHz). All instruments were operated by the Core Facility BioSupraMol of the Freie Universität Berlin, too. Data processing was performed with MestReNova (*v*6.0.2-5475).

Quantification of Regioisomer Proportions

Relative regioisomer proportions of [pG1]-OH, *e.g.*, (**a**:**b**), were analyzed by means of analytical normal phase (NP) HPLC. For NP HPLC analysis, a Nucleosil column from Macherey Nagel was used as a stationary phase (pore size: 50 Å, particle size: 5 µm, length: 250 mm, diameter: 4 mm), while mixtures of *n*-hexane and isopropanol were used (*v*:*v*) as a mobile phase. The NP HPLC system was equipped with a smartline pump 1050, a smartline UV detector 2550, and a smartline RI detector 2300, which were purchased from Knauer. Data processing and analysis were performed with ChromeGate Client Viewer (*v*.3.3.2) from Knauer. The NP HPLC system was operated by Marleen Selent. Regioisomer proportions of other [pG1]-based mixtures were characterized by ^{1}H or ^{13}C NMR. To determine their relative regioisomer proportions the relative intensities of the focal point signals were extracted from the NMR spectra. For this purpose, relevant ^{13}C NMR spectra were acquired using the inverse-gated mode and the sample concentrations in deuterated solvents were about 180 mg/mL.

Regioisomer mixtures of [G1] OGD mixtures **1** – **2** were analyzed by analytical RP HPLC. Experiments were performed in isocratic mode with a system from Knauer, equipped with two Smartline 1000 pumps, variable wavelength UV detector 2500, and an Autosampler 3950. As stationary phase a pre-packed Kinetex EVO C18 column was used (pore size: 100 Å, particle size: 5 µm, length: 250 mm, diameter: 4.6 mm), purchased from Phenomenex. Degassed mixtures of H_2O and MeOH were used (*v*:*v*) and thermally equilibrated upon mixing for at least 12 hours prior to use. Data processing and analysis was performed with a ChromeGate Client Viewer (*v*.3.3.2) from Knauer.

Relative regioisomer proportions of [pG2]-based derivatives and [G2] OGD mixtures **3** – **5**, *e.g.*, (**aa**:**ab**:**bb**), were analyzed by ^{13}C NMR as described above. The sample concentrations in deuterated solvents were about 200 – 300 mg/mL.

Synthesis of [pG1]-OH (a, b)

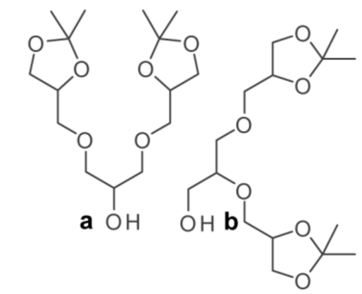

Technical triglycerol (Fluka, product number: 17782) was dissolved in 2,2´-dimethoxypropane (3 eq.) and PTSA*H_2O (0.1 eq.) was added. The reaction mixture was stirred at 40 °C for 16 h. NEt_3 (0.1 eq.) was added and the solvent was removed under reduced pressure. The raw material was purified by column chromatography (SiO_2, *n*-hexane/EtOAc, 2:1 → 1:6) and the product [pG1]-OH was obtained as pale yellow oil (**a**:**b**, 6:4, 60%). ^{1}H NMR (400 MHz, [D_4] MeOH) δ = 4.28 - 4.20 (m, 2H), 4.06 - 3.99 (m, 2H), 3.87 - 3.80 (m, 1H), 3.75 - 3.44 (m, 10 H), 1.40 - 1.30 (m, 12H). ^{13}C NMR (101 MHz, [D_4] MeOH) δ = 110.4, 81.4, 76.3 - 76.1, 73.9, 73.4, 72.4 - 72.3, 70.6, 67.5, 62.6, 27.0, 25.6. MS (ESI): *m/z* = 343.1739 $C_{15}H_{28}O_7Na_1^+$ (calc. 343.1727).

Synthesis of [pG1]-ether-C12 (a, b)

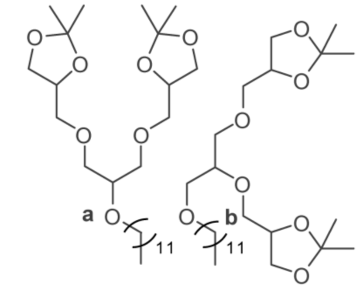

[pG1]-OH (8.00 g, 24.9 mmol, **a**:**b**, 6:4) was dried under reduced pressure (~ 10^{-2} mbar) and DMF (200 mL) was added. The flask was cooled with an ice bath and NaH (60w%, 6.24 g, 156 mmol) was added in small portions. The ice bath was removed and the mixture was allowed to warm up to RT. 12-Bromododecane (31.1 g, 124 mmol) was added slowly and the mixture was stirred for 16 h. The mixture was again cooled with an ice bath and a saturated solution of NH_4Cl (100 mL) was added slowly. The solvent was removed under reduced pressure. The residue was suspended with H_2O (200 mL), brine (200 mL), and EtOAc (200 mL). The aqueous layer was extracted with EtOAc (4 x 200 mL). The organic layer was dried over Na_2SO_4. The solvent was removed under reduced pressure and subsequent column chromatography (SiO_2, pentane/EtOAc, 3:1 → 1:1) gave the desired product [pG1]-ether-C12 (12.1 g, 24.8 mmol, **a**:**b**, 99%). ^{1}H NMR (400 MHz, [D_4] MeOH): δ = 4.28 - 4.19 (m, 2H), 4.08 - 4.01 (m, 2H), 3.79 - 3.40 (m, 13H), 1.60 - 1.49 (m, 2H), 1.42 - 1.23 (m, 30H), 0.94 - 0.87 (m, 3H). ^{13}C NMR (101 MHz, [D_4] MeOH): δ = 110.4, 79.1, 76.1, 73.3, 72.4 - 72.3, 71.4, 67.6, 31.6, 30.8 - 30.5, 27.1, 25.7, 23.7, 14.5. MS (ESI): *m/z* = 511.3609 $C_{27}H_{52}O_7Na_1^+$ (calc. = 511.3605).

Synthesis of [G1]-ether-C12 (a, b) – 1

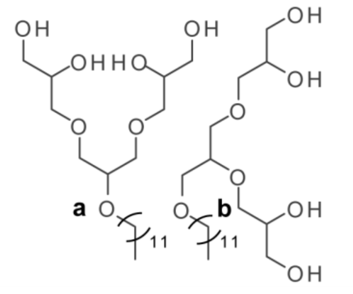

[pG1]-ether-C12 (12.0 g, 24.8 mmol, **a**:**b**) was dissolved in MeOH (1 L) and HCl (37%, 100 µL) was added. The mixture was stirred for 12 h at RT and the solvent was removed under reduced pressure. The residue was again dissolved and in MeOH (1 L) and HCl (37%, 100 µL) was added. The solvent was removed under reduced pressure. The crude material was dissolved in a mixture of H_2O and MeOH (1:1, 70 mL) and passed through a syringe filter (RC, 0.2 µm). Purification by RP HPLC (H_2O/MeOH, 3:7) gave the desired product (8.00 g, 19.6 mmol, **a**:**b** , 6:4, 80%). ^{1}H NMR (500 MHz, [D_4] MeOH): δ = 3.80 - 3.71 (m, 2H), 3.64 - 3.44 (m, 15H), 1.60 - 1.53 (m, 2H), 1.41 - 1.25 (m, 18H), 0.93 - 0.86 (m, 3H). ^{13}C NMR (126 MHz, [D_4] MeOH): δ = 77.8, 72.6 - 72.5, 71.3 - 70.7, 70.2 - 70.0, 63.1 - 63.0, 31.7, 29.7 - 29.1, 25.8, 22.4, 13.3 - 13.1. MS (ESI): *m/*z = 431.2996 $C_{21}H_{44}O_7Na_1^+$ (calc. = 431.2979).

Synthesis of [pG1]-O-propargyl (a, b)

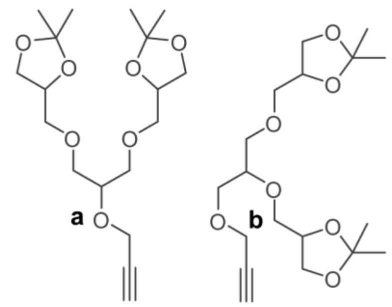

[pG1]-OH (2.50 g, 7.80 mmol, **a**:**b**, 6:4) was first dried under reduced pressure (~ 10^{-2} mbar) and then dissolved in DMF (70 mL). The mixture was cooled with an ice bath and NaH (60w%, 936 mg, 39.0 mmol) was added in small portions. The ice bath was removed and the mixture was allowed to warm up to RT. Catalytic amounts of 15-crown-5 and propargyl bromide (80w%, 4.35 mL, 39.0 mmol) were added. The mixture was stirred for 12 h and a saturated solution of NH_4Cl (50 mL) was added. The solvent was removed under reduced pressure and the residue was treated with H_2O (200 mL), EtOAc (150 mL), and brine (100 mL). The aqueous layer was extracted with EtOAc (3 x 150 mL), the organic layer was dried over Na_2SO_4, and the solvent was removed under reduced pressure. Column chromatography (SiO_2, pentane/EtOAc, 9:1 → 1:1) gave the desired product (2.10 g, 5.85 mmol, 75%, **a**:**b**, 6:4). ^{1}H NMR (400 MHz, [D_4] MeOH): δ = 4.33 - 4.30 (m, 1.2H), 4.29 - 4.20 (m, 2H), 4.19 - 4.17 (m, 0.8H), 4.07 - 4.02 (m, 2H), 3.88 - 3.48 (m, 11H), 2.89 - 2.85 (m, 1H), 1.41 - 1.31 (m, 12H). ^{13}C NMR (101 MHz, [D_4] MeOH): δ = 110.4 - 110.3, 81.1, 80.6, 79.6, 77.7, 76.1 - 76.0, 75.7, 73.3 - 73.2, 72.4 - 72.2, 70.5, 67.7, 67.5, 59.2, 58.3 - 58.2, 27.1 - 27.0, 25.7 - 25.6. MS (ESI): *m/*z = 381.1890 $C_{18}H_{30}O_7Na_1^+$ (calc. = 381.1884).

Synthesis of [pG1]-triazole-C12 (a, b)

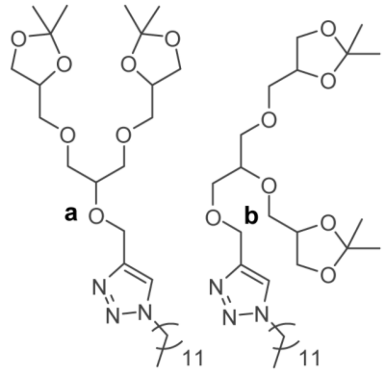

[pG1]-O-propargyl (2.00 g, 5.58 mmol, **a**:**b**, 6:4) and 1-azidododecane (1.29 g, 6.14 mmol) were dissolved in THF (5 mL) and H_2O (2 mL) was added. DIPEA (94.9 µL, 0.56 mmol), sodium ascorbate (552 mg, 2.79 mmol, dissolved in 2 mL H_2O), and Cu(II)SO_4*5H_2O (139 mg, 0.56 mmol, dissolved in 1 mL H_2O) were added. The mixture was stirred at RT for 12 h and then diluted with H_2O (40 mL). A saturated solution of EDTA (1 mL) and brine (10 mL) was added. The aqueous layer was extracted with EtOAc (3 x 40 mL) and the solvent was removed under reduced pressure. Column chromatography (SiO_2, DCM/EtOAc, 4:1 → 4:1 + 3% MeOH) gave the desired product (2.22 g, 3.80 mmol, **a**:**b**, 68%). ^{1}H NMR (400 MHz, [D_4] MeOH): δ = 8.03 (s, 1H), 4.80 - 4.58 (m, 2H), 4.44 - 4.36 (m, 2H), 4.27 - 4.17 (m, 2H), 4.07 - 3.98 (m, 2H), 3.75 - 3.45 (m, 11H), 1.95 - 1.86 (m, 2H), 1.39 - 1.24 (m, 30H), 0.93 - 0.86 (m, 3H).^{13}C NMR (101 MHz, [D_4] MeOH): δ = 110.5 - 110.2, 79.8 - 79.7, 78.6 - 78.5, 76.1 - 76.0, 73.6 - 73.3, 72.5 - 72.3, 71.1, 51.3, 33.0, 31.3, 30.7 - 30.1, 27.4, 27.1, 25.7 - 25.6, 23.7, 14.5. MS (ESI): *m/*z: 592.3982 $C_{30}H_{55}N_3O_7Na_1^+$ (calc. = 592.3932).

Synthesis of [G1]-triazole-C12 (a, b) – 2

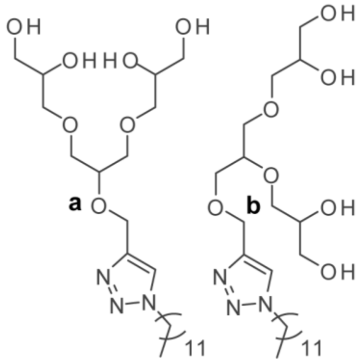

[pG1]-triazole-C12 (2.22 g, 3.80 mmol, **a**:**b**) was dissolved in MeOH (500 mL) and HCl (37%, 100 µL) was added. The mixture was stirred at RT for 12 h and solvent was removed under reduced pressure. The material was again dissolved in MeOH (500 mL) and HCl (37%, 100 µL) was added. Upon stirring for 12 h at RT the solvent was removed under reduced pressure. The crude material was dissolved in a mixture of H_2O and MeOH (1:1, 20 mL), passed through a syringe filter (RC, 0.2 µm), and purified by means of RP HPLC (H_2O/MeOH, 3:7) to give the desired product (1.48 g, 3.04 mmol, **a**:**b**, 6:4, 80%). ^{1}H NMR (400 MHz, [D_4] MeOH): δ = 8.06 - 7.99 (m, 1H), 4.81 - 4.61 (m, 2H), 4.43 - 4.35 (m, 2H), 3.87 - 3.43 (m, 15H) 1.96 - 1.83 (m, 2H), 1.41 - 1.20 (m, 18H), 0.93 - 0.85 (m, 3H). ^{13}C NMR (101 MHz, [D_4] MeOH): δ = 146.1 - 145.6, 125.0 - 124.9, 73.8, 72.7, 72.3 - 72.2, 72.0, 71.1, 65.1, 64.2, 64.1, 51.2, 32.9, 31.2, 30.6 - 30.4, 30.0, 27.4, 23.6, 14.5. MS (ESI): *m/*z = 512.3357 $C_{24}H_{47}N_3O_7Na_1^+$ (calc. = 512.3306).

Synthesis of [pG2]-ene (aa, ab, bb)

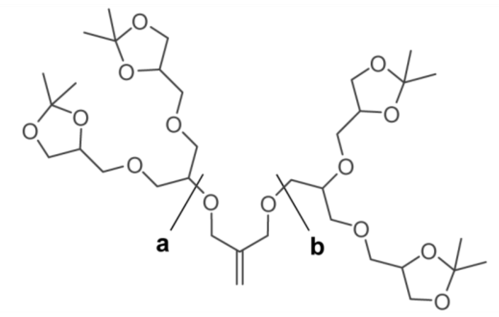

[pG1]-OH (13.6 g, 42.5 mmol, **a**:**b**, 6:4) was dried under reduced pressure (~ 10^{-2} mbar), dissolved in dry THF (125 mL), and NaH (60w%, 5.10 g, 128 mmol) was added in small portions. Catalytic amounts of 15-crown-5 were added and the mixture was stirred for 45 min at 50 °C. Methallyl dichloride (2.46 mL, 21.2 mmol), catalytic amounts of potassium iodide, and catalytic amounts of 18-crown-6 were added. The reaction was stirred at 70 °C for 24 h. The mixture was then cooled with an ice bath and H_2O (80 mL) was slowly added. The solvent was removed under reduced pressure and the crude product was mixed with brine (200 mL) and DCM (150 mL). The aqueous layer was extracted with DCM (3 x 150 mL). The organic layer was dried over Na_2SO_4 and the solvent was removed under reduced pressure. Column chromatography (SiO_2, pentane/EtOAc, 1:2 → 0:1) gave the desired product (12.2 g, 17.6 mmol, **aa**:**ab**:**bb**, 4:4:1, 83%).^{1}H NMR (400 MHz, [D$_4$] MeOH): δ = 5.22 - 2.21 (m, 2H), 4.29 - 4.22 (m, 4H), 4.19 - 4.13 (m, 2H), 4.08 - 4.01 (m, 4H), 3.79 - 3.46 (m, 24 H), 1.40 - 1.30 (m, 24H). ^{13}C NMR (101 MHz, [D$_4$] MeOH): δ = 145.2, 144.8, 114.5, 110.4, 79.9, 78.4, 76.1, 73.4, 72.9, 72.4 - 72.3, 71.6, 71.1, 67.7 - 67.6, 27.1, 25.7. MS (ESI): *m/z* = 715.3982 $C_{34}H_{60}O_{14}Na_1^+$ (calc. = 715.3875).

Synthesis of [pG2]-OH (aa, ab, bb)

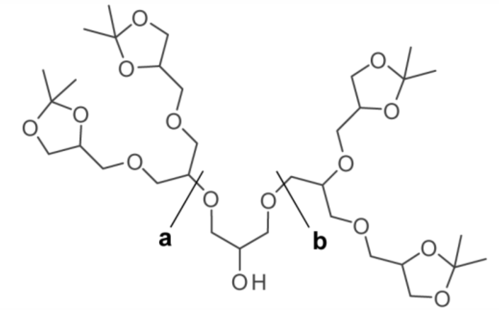

[pG2]-ene (8.00 g, 11.5 mmol, **aa**:**ab**:**bb**, 4:4:1) was dried was dried under reduced pressure (~ 10^{-2} mbar) and dissolved in a mixture of dry DCM (35 mL) and dry MeOH (35 mL). The mixture was cooled down to - 78 °C and ozone was passed through the reaction mixture until its color changed to deep blue. Oxygen was then passed through the solution until it became colorless. Sodium borohydride (4.36 g, 115 mmol) was added slowly and the mixture was allowed to warm up to RT overnight. A saturated solution of NH_4Cl (100 mL) was added and the mixture was extracted with DCM (5 x 50 mL). The organic layer was dried over Na_2SO_4 and the solvent was removed under reduced pressure. Column chromatography (SiO_2, DCM/EtOAc, 97:3 + 3% MeOH) gave the desired product (6.40 g, 9.18 mmol, **aa**:**ab**:**bb**, 4:4:1, 80%). ^{1}H NMR (400 MHz, [D$_4$] MeOH): δ = 4.29 - 4.21 (m, 4H), 4.08 - 4.02 (m, 4H), 3.80 - 3.47 (m, 27H), 1.42 - 1.31 (m, 24H). ^{13}C NMR (101 MHz, [D$_4$] MeOH): δ = 109.1, 78.5, 74.8, 72.5 - 72.1, 71.4 - 71.1, 69.8 - 69.5, 66.2, 25.8, 24.3. MS (ESI): *m/*z = 719.3920 $C_{33}H_{60}O_{15}Na_1^+$ (calc. = 719.3824).

Synthesis of [pG2]-ether-C18 (aa, ab, bb)

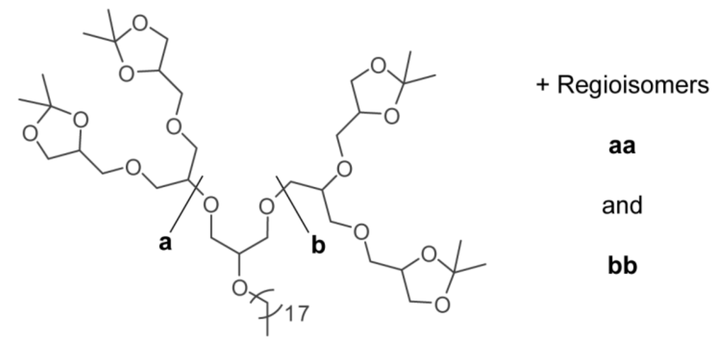

[pG2]-OH (4.80 g, 6.88 mmol, **aa**:**ab**:**bb**, 4:4:1) was dried under reduced pressure (~ 10^{-2} mbar) and dissolved in DMF (120 mL). NaH (60w%, 1.38 g, 34.4 mmol) was added in small portions and 1-bromooctadecane (11.5 g, 34.4 mmol) was added subsequently. The mixture was stirred at 80 °C for 16 h. The mixture was cooled with an ice bath and a saturated solution of NH_4Cl (50 mL) was added slowly. The solvent was removed under reduced pressure, H_2O (200 mL) was added, the aqueous layer was extracted with EtOAc (3 x 200 mL). Column chromatography (SiO_2, pentane/EtOAc, 1:1 → 1:1 + 2% MeOH) gave the desired product (4.45 g, 4.69 mmol, **aa**:**ab**:**bb**, 68%). ^{1}H NMR (400MHz, [D$_4$] MeOH): δ = 4.28 - 4.20 (m, 4H), 4.08 - 4.02 (m, 4H), 3.79 - 3.47 (m, 29H), 1.61 - 1.52 (m, 2H), 1.44 - 1.23 (m, 54H), 0.95 - 0.88 (m, 3H). ^{13}C NMR (101 MHz, [D$_4$] MeOH): δ = 110.4, 79.9 - 79.8, 76.2 - 76.0, 73.4, 72.4, 71.3 - 71.1, 67.8 - 67.6, 33.1, 31.2 - 30.5, 27.3 - 27.2,. 25.7, 23.7, 14.5. MS (ESI): *m/*z = 971.6591 $C_{51}H_{96}O_{15}Na_1^+$ (calc. = 971.6641).

Synthesis of [G2]-ether-C18 (aa, ab, bb) – 3

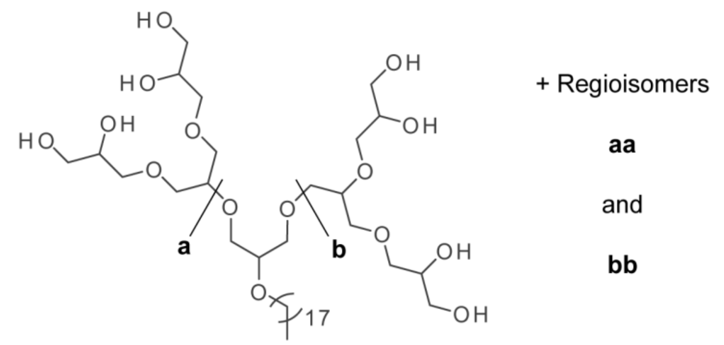

[pG2]-ether-C18 (4.40 g, 4.63 mmol, **aa**:**ab**:**bb**) was dissolved in MeOH (500 mL) and three tablespoons full with Amberlite® IR-120(H) were added. The mixture was stirred vigorously for 16 h, Amberlite® IR-120(H) was filtered off, the solvent was removed under reduced pressure, and the procedure was repeated. The crude product was dissolved in a mixture of H_2O and MeOH (1:1, 38 mL), passed through a syringe filter (RC, 0.2 µm) and purification by means of RP HPLC (H_2O/MeOH, 1:9) gave the desired product (3.20 g, 4.06 mmol, **aa**:**ab**:**bb**, 4:4:1, 88%). ^{1}H NMR (400 MHz, [D$_4$] MeOH): δ = 3.79 - 3.46 (m, 37H), 1.61 - 1.51 (m, 2H), 1.38 - 1.26 (m, 30H), 0.94 - 0.88 (m, 3H). ^{13}C NMR (101 MHz, [D$_4$] MeOH): δ = 79.8 - 79.3, 73.9, 72.9, 72.4 - 72.1, 71.4, 70.9, 64.4 - 64.3, 33.0, 31.1, 30.8 - 30.6, 30.4, 27.2, 23.7, 14.4. MS (ESI): *m/*z = 811.5476 $C_{39}H_{80}O_{15}Na_1^+$ (calc. = 811.5389).

Synthesis of [pG2]-N_3 (aa, ab, bb)

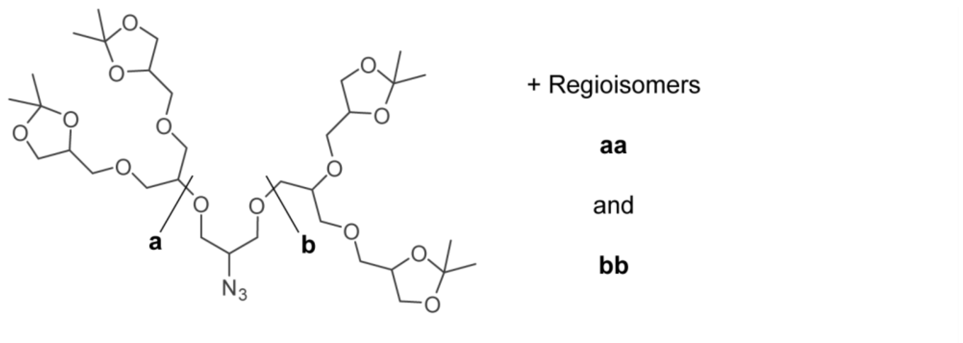

[pG2]-OH (1.50 g, 2.15 mmol, **aa**:**ab**:**bb**, 4:4:1) was dissolved in toluene (100 mL), NEt_3 (0.41 mL, 3.22 mmol) was added, and MsCl (0.22 mL, 2.78 mmol) was added. The mixture was stirred for 16 h at RT, the precipitate was filtered off, and the solvent was removed under reduced pressure. The obtained raw material was dissolved in DMF (100 mL), NaN_3 (0.70 g, 10.7 mmol) was added, and the mixture was stirred for 2 h at 120 °C. The excess of NaN_3 was filtered off, the solvent was removed under reduced pressure, and column purification (SiO_2, DCM/EtOAc, 4:1 → 3:1) gave the desired product (0.98 g, 1.35 mmol, **aa**:**ab**:**bb**, 3:4:1, 63%). ^{1}H NMR (700 MHz, [D_4] MeOH): δ = 4.27 - 4.22 (m, 4H), 4.08 - 4.02 (m, 4H), 3.80 - 3.31 (m, 27H), 1.47 - 1.28 (m, 24H). ^{13}C NMR (175 MHz, [D_4] MeOH): δ = 110.4, 82.3 - 82.0, 79.9 - 79.8, 76.1 - 76.0, 73.4, 72.5 - 72.1, 71.7, 71.1, 70.6, 67.4 - 67.5, 62.4 - 61.9, 38.1, 27.1, 25.7.

Synthesis of [pG2]-NH_2 (aa, ab, bb)

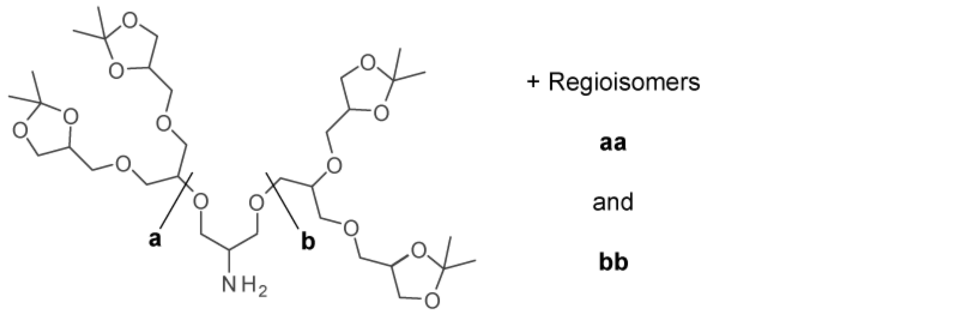

[pG2]-N_3 (1.30 g, 1.80 mmol, **aa**:**ab**:**bb**, 3:4:1) was dissolved in MeOH (10 mL) and Pd/C (100 mg) was added, and the mixture was stirred under hydrogen atmosphere (5 bar) for 16 h. The mixture was passed subsequently through a syringe filter (0.2 µm, RC) and the solvent was removed under reduced pressure to obtain the desired product (1.00 g, 1.43 mmol, **aa**:**ab**:**bb**, 4:4:1, 80%). ^{1}H NMR (700 MHz, [D_4] MeOH): δ = 4.65 (s, 2H), 4.27 - 4.21 (m, 4H), 4.06 - 4.04 (m, 4H), 3.89 - 3.77 (m, 27H), 1.46 - 1.25 (m, 24H). ^{13}C NMR (175 MHz, [D_4] MeOH): δ = 110.3, 82.2, 81.9, 79.9 - 79.6, 76.1 - 76.0, 73.8 - 73.7, 73.3, 72.7 - 72.1, 71.6 - 71.5, 70.5, 67.6 - 67.5, 52.2 - 51.6, 27.1, 25.7.

Synthesis of [pG2]-carbamate-Chol (aa, ab, bb)

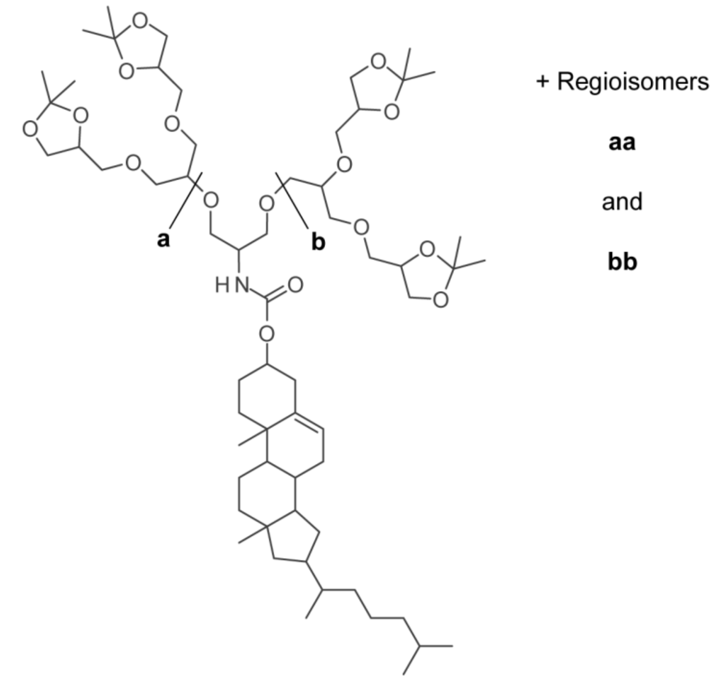

[pG2]-NH_2 (1.00 g, 1.44 mmol, **aa**:**ab**:**bb**, 4:4:1) was dried under reduced pressure (~ 10^{-2} mbar). Dry DCM (40 mL), NEt_3 (1.00 mL, 7.18 mmol), and cholic acid chloride (2.60 g, 5.78 mmol) were added. The mixture was stirred at RT for 12 h. Column chromatography (SiO_2, DCM/EtOAc, 4:1 → 1:1) led to the obtainment of the desired product (1.19 g, 1.06 mmol, **aa**:**ab**:**bb**, 74%). ^1H NMR (700 MHz, [D_2] DCM): δ = 5.42 - 5.41 (m, 1H), 4.48 - 4.45 (m, 1H), 4.29 - 4.92 (m, 4H), 4.07 - 4.04 (m, 4H), 3.75 - 3.47 (m, 27 H), 2.38 - 0.98 (m, 29H), 1.41 (s, 12H), 1.36 (s, 12H), 1.05 (s, 3H), 0.96 (m, 3H), 0.90 (m, 6H), 0.72 (m, 3H). ^{13}C NMR (176 MHz, [D_2] DCM): δ = 156.0, 140.4, 122.7, 109.6, 79.3, 79.1, 78.9, 75.3, 75.1, 75.0, 74.6, 72.9, 71.9, 71.8, 71.7, 71.5, 70.6, 70.5, 69.6, 69.4, 67.2, 67.1, 57.1, 56.5, 50.5, 42.7, 40.2, 39.9, 39.0, 37.4, 36.9, 36.6, 36.2, 32.3, 28.6, 28.4, 26.9, 25.6, 24.6, 24.2, 22.9, 22.7, 21.4, 19.5, 18.9, 12.0. MS (ESI): *m/z* = 1130.7350 $C_{61}H_{105}N_1O_{16}Na_1^+$ (calc. = 1130.7326).

Synthesis of [G2]-carbamate-Chol (aa, ab, bb) – 4

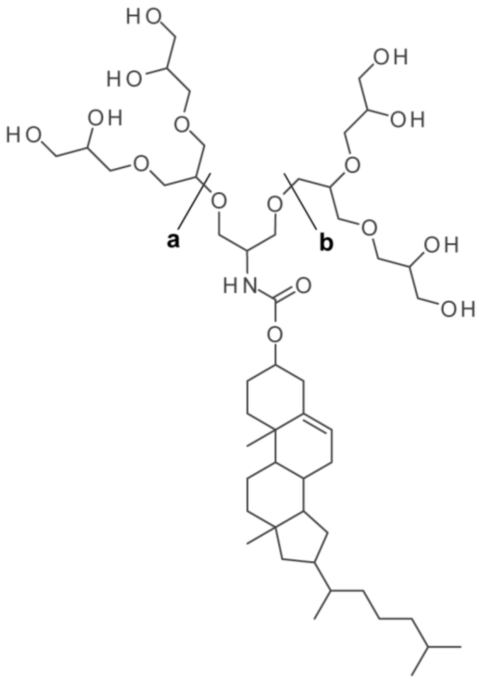

[pG2]-carbamate-Chol (1.18 g, 1.06 mmol, **aa**:**ab**:**bb**) was dissolved in MeOH (50 mL) and two tablespoons full with Amberlite® IR-120(H) were added. The mixture was stirred at RT for 16 h and Amberlite® IR-120(H) was filtered off. The solvent was removed under reduced pressure, the residue was dissolved in a mixture of H_2O and MeOH (1:1, 20 mL), and the solution was passed through a syringe filter (RC, 0.2 µm). Purification by RP HPLC (H_2O/MeOH, 5:95) gave the desired product (0.70 g, 0.74 mmol, **aa**:**ab**:**bb**, 9:5:1, 70%). ^{1}H NMR (700 MHz, [D_1] $CHCl_3$): δ = 5.38 - 5.37 (m, 1H), 4.43 - 4.35 (m, 1H), 3.79 - 3.74 (m, 4H), 3.71 - 3.66 (m, 5H), 3.62 - 3.46 (m, 26H), 2.33 - 1.07 (m, 29H), 1.02 (s, 3H), 0.93 (m, 3H), 0.87 (m, 6H), 0.71 (s, 3H). ^{13}C NMR (126 MHz, [D_1] $CHCl_3$): δ = 158.2, 141.2, 123.4, 101.7, 79.8, 73.9, 72.3, 72.1, 64.4, 58.1, 57.5, 51.6, 43.5, 42.3, 41.1, 40.6, 39.6, 38.2, 37.7, 37.3, 37.1, 36.9, 33.2, 33.0, 29.3, 29.1, 27.4, 25.3, 24.9, 23.2, 22.9, 22.1, 19.8, 19.2, 12.3. MS (ESI): *m/z* = 970.6047 $C_{49}H_{89}N_1O_{16}Na_1^+$ (calc. = 970.6074).

Synthesis of [pG2]-O-propargyl (aa, ab, bb)

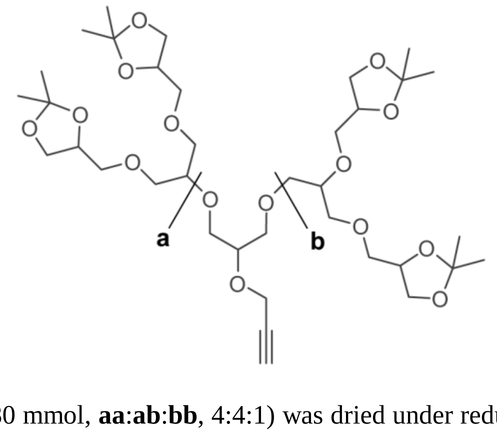

[pG2]-OH (2.00 g, 2.80 mmol, **aa**:**ab**:**bb**, 4:4:1) was dried under reduced pressure (~ 10^{-2} mbar) and dissolved in DMF (70 mL). The flask was cooled with an ice bath and NaH (60w%, 0.35 g, 14.4 mmol) was added in small portions. The ice bath was removed and the mixture was heated up to 60 °C for 30 min. The temperature was reduced to 40 °C, catalytic amounts of 15-crown-5, and propargyl bromide (80w%, 1.56 mL, 14.0 mmol) were added. The mixture was stirred at 40 °C for 12 h before a saturated solution of NH_4Cl (50 mL) was slowly added. The solvent was removed under reduced pressure and the residue was mixed with H_2O (200 mL), EtOAc (150 mL), and brine (100 mL). The aqueous layer was extracted with EtOAc (3 x 150 mL), the organic layer was dried over Na_2SO_4, and the solvent was removed under reduced pressure. Column chromatography

(SiO_2, pentane/EtOAc, 3:1 → 3:1 + 2% MeOH) gave the desired product (1.60 g, 2.17 mmol, **aa**:**ab**:**bb**, 4:4:1, 78%). ^{1}H NMR (400 MHz, [D_4] MeOH): δ = 4.35 - 4.31 (m, 2H), 4.28 - 4.21 (m, 4H), 4.08 - 4.02 (m, 4H), 3.79 - 3.50 (m, 27H), 2.89 - 2.85 (m, 1H), 1.41 - 1.29 (m, 24H). ^{13}C NMR (101 MHz, [D_4] MeOH): δ = 110.3, 81.3 - 81.2, 79.8 - 79.7, 78.4 - 77.7, 76.7 - 75.7, 73.3, 72.4 - 72.3, 71.1, 67.7 - 67.5, 58.2, 27.2 - 27.1, 25.7. MS (ESI): *m/*z = 757.4073 $C_{36}H_{62}O_{15}Na_1^+$ (calc. = 757.3981).

Synthesis of [pG2]-triazole-DC12 (aa, ab, bb)

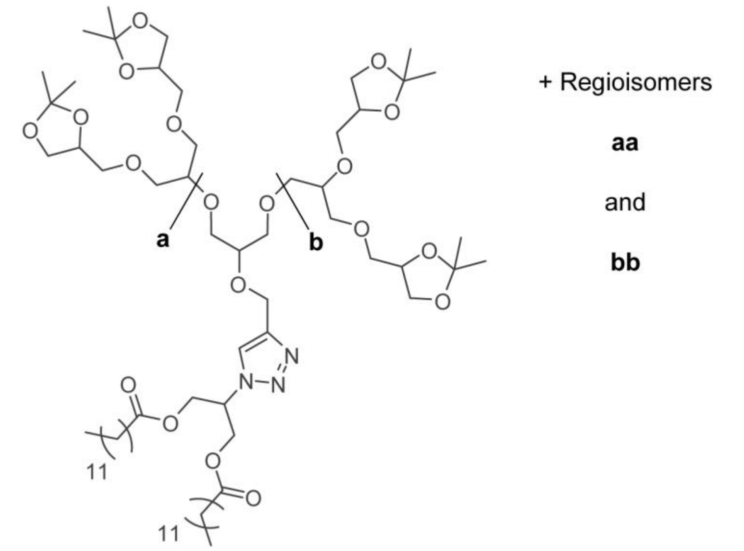

[pG2]-O-propargyl (0.73 g, 1.00 mmol, **aa**:**ab**:**bb**, 4:4:1) and dodecanoic acid, 2-azido-1,3-propanediyl ester (0.76 g, 1.50 mmol) were dissolved in THF (3 mL) and H_2O (3 mL). DIPEA (17.5 µL, 0.10 mmol), sodium ascorbate (99.5 mg, 0.50 mmol dissolved in 1 mL H_2O) and Cu(II)SO_4*5H_2O (24.9 mg, 0.10 mmol dissolved in 1 mL H_2O) were added and the mixture was stirred at RT for 2 h. A saturated solution of EDTA (1 mL) and brine (20 mL) was added. The aqueous layer was extracted with EtOAc (3 x 50 mL), the organic layer was dried over Na_2SO_4, and the solvent was removed under reduced pressure. Column chromatography (SiO_2, pentane/isopropanol, 9:1) gave the desired product (0.88 g, 0.72 mmol, **aa**:**ab**:**bb**, 72%). ^{1}H NMR (400 MHz, [D_4] MeOH): δ = 8.11 (m, 1H), 5.23 - 5.17 (m, 1H), 4.82 - 4.79 (m, 2H), 4.62 - 4.53 (m, 4H), 4.27 - 4.21 (m, 4H), 4.07 - 4.00 (m, 4H), 3.79 - 3.46 (m, 27H), 2.33 - 2.27 (m, 4H), 1.60 - 1.50 (m, 4H), 1.44 - 1.18 (m, 56H), 0.94 - 0.87 (m, 6H). ^{13}C NMR (101 MHz, [D_4] MeOH): δ = 174.3, 110.4, 79.8, 76.1, 73.4, 72.4, 67.6, 63.4, 34.6, 33.0, 30.7 - 30.4, 30.1, 27.1, 25.8, 25.7, 23.7, 14.5.

Synthesis of [G2]-triazole-DC12 (aa, ab, bb) – 5

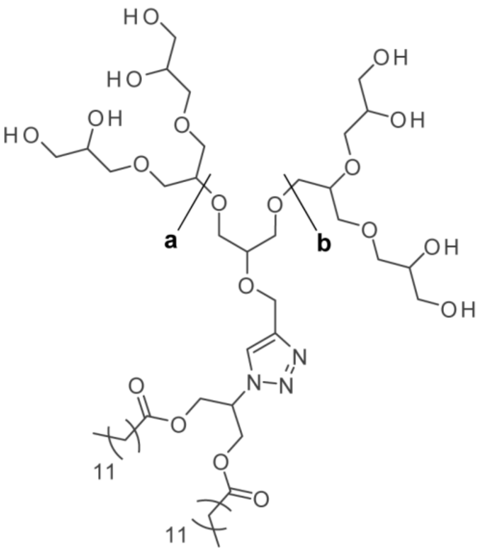

[pG2]-triazole-DC12 (0.88 g, 0.72 mmol, **aa**:**ab**:**bb**) was dissolved in MeOH (30 mL), two tablespoons full with Amberlite® IR-120(H) were added, and the mixture was heated up to 40 °C for 6 h. Amberlite® IR-120(H) was filtered off and the solvent was removed under reduced pressure. The crude product was dissolved in a mixture of H_2O and MeOH (1:1, 15 mL) and the solution was passed through a syringe filter (RC, 0.2 µm). Purification by RP HPLC (H_2O/MeOH, 1:9) gave the desired product (0.45 g, 0.42 mmol, **aa**:**ab**:**bb**, 9:5:1, 58%).

^{1}H NMR (400 MHz, [D$_4$] MeOH): 8.16 (m, 1H), 5.23 - 5.18 (m, 1H), 4.81 - 4.77 (m, 2H), 4.61 - 4.54 (m, 4H), 3.81 - 3.45 (m, 35H), 2.32 - 2.29 (m, 4H), 1.59 - 1.48 (m, 4H), 1.34 - 1.22 (m, 32H), 0.93 - 0.87 (m, 6H). δ = ^{13}C NMR (101 MHz, [D$_4$] MeOH): δ = 174.5, 146.3, 124.9, 79.9 - 79.2, 74.0 - 73.9, 72.9, 72.4 - 72.1, 71.2, 64.4, 64.2, 63.4, 60.3, 34.6, 33.0, 30.7 - 30.4, 30.1, 25.8, 23.7, 14.4. MS (ESI): *m/z* = 1078.6623 $C_{51}H_{97}N_3O_{19}Na_1^+$ (calc. = 1078.6608).

4. Supplementary References

1. Kramer, R. A., Zandwijken, D., Egmond, M. R., Dekker, N. In vitro folding, purification and characterization of Escherichia coli outer membrane protease OmpT. *Eur. J. Biochem.* **267**, 885-893 (2000).

2. Reading, E., Liko, I., Allison, T. M., Benesch, J. L. P., Laganowsky, A., Robinson, C. V. The Role of the Detergent Micelle in Preserving the Structure of Membrane Proteins in the Gas Phase. *Angew. Chem. Int. Ed.* **54**, 4577-4581 (2015).

3. Urner, L. H., Maier, Y. B., Haag, R., Pagel, K. Exploring the Potential of Dendritic Oligoglycerol Detergents for Protein Mass Spectrometry. *J. Am. Soc. Mass Spectrom.* **30**, 174-180 (2018).

4. Laganowsky, A., Reading, E., Allison, T. M., Ulmschneider, M. B., Degiacomi, M. T., Baldwin, A. J., Robinson, C. V. Membrane proteins bind lipids selectively to modulate their structure and function. *Nature.* **510**, 172-175 (2014).

5. Calabrese, A. N., Watkinson, T. G., Henderson, P. J. F., Radford, S. E., Ashcroft, A. E. Amphipols Outperform Dodecylmaltoside Micelles in Stabilizing Membrane Protein Structure in the Gas Phase. *Anal. Chem.* **87**, 1118-1126 (2015).

6. Urata, K., Takaishi, N., Suzuki, Y. Neue Polyolether-Verbindungen, Verfahren zur Herstellung derselben und Kosmetika mit einem Gehalt derselben. DE 3427093A3427091 (1983).

7. Thota, B. N. S., v. Berlepsch, H., Böttcher, C., Haag, R. Towards engineering of self-assembled nanostructures using non-ionic dendritic amphiphiles. *Chem. Commun.* **51**, 8648 - 8651 (2015).

8. Haag, R., Wyszogrodzka, M., Wiedekind, A., Mohr, A., Rehage, H., Trappmann, B. Linear-dendritische Polyglycerol-verbindungen, Verfahren zu ihrer Herstellung und ihre Verwendung. DE 10 2008 030992A030991 (2008).

9. Wyszogrodzka, M., Haag, R. A Convergent Approach to Biocompatible Polyglycerol "Click" Dendrons for the Synthesis of Modular Core–Shell Architectures and Their Transport Behavior. *Chem. Eur. J.* **14**, 9202 - 9214 (2008).

10. Huth, K., Heek, T., Achazi, K., Kühne, C., Urner, L. H., Pagel, K., Dernedde, J., Haag, R. Noncharged and Charged Monodendronised Perylene Bisimides as Highly Fluorescent Labels and their Bioconjugates. *Chem. Eur. J.* **23**, 4849-4862 (2017).
