# Supplementary material for: Modular detergents tailor the purification and structural analysis of membrane proteins including G-protein coupled receptors
Source: Nat Commun. 2020 Jan 28;11:564. doi: 10.1038/s41467-020-14424-8 (PMC6987200; doi:10.1038/s41467-020-14424-8)
Supplement: Supplementary file 3 — Reporting Summary [file 41467_2020_14424_MOESM3_ESM.pdf]

nature research

Corresponding author(s): Rainer Haag, Carol V. Robinson, Kevin Pagel

Last updated by author(s): Dec 16, 2019

Reporting Summary

Nature Research wishes to improve the reproducibility of the work that we publish. This form provides structure for consistency and transparency in reporting. For further information on Nature Research policies, see Authors & Referees and the Editorial Policy Checklist .

Statistics

For all statistical analyses, confirm that the following items are present in the figure legend, table legend, main text, or Methods section.

n/a	Confirmed	
☐	☒	The exact sample size (n) for each experimental group/condition, given as a discrete number and unit of measurement
☐	☒	A statement on whether measurements were taken from distinct samples or whether the same sample was measured repeatedly
☐	☒	The statistical test(s) used AND whether they are one- or two-sided<br>*Only common tests should be described solely by name; describe more complex techniques in the Methods section.*
☒	☐	A description of all covariates tested
☒	☐	A description of any assumptions or corrections, such as tests of normality and adjustment for multiple comparisons
☐	☒	A full description of the statistical parameters including central tendency (e.g. means) or other basic estimates (e.g. regression coefficient) AND variation (e.g. standard deviation) or associated estimates of uncertainty (e.g. confidence intervals)
☐	☒	For null hypothesis testing, the test statistic (e.g. F, t, r) with confidence intervals, effect sizes, degrees of freedom and P value noted<br>*Give P values as exact values whenever suitable.*
☒	☐	For Bayesian analysis, information on the choice of priors and Markov chain Monte Carlo settings
☒	☐	For hierarchical and complex designs, identification of the appropriate level for tests and full reporting of outcomes
☒	☐	Estimates of effect sizes (e.g. Cohen's d, Pearson's r), indicating how they were calculated

Our web collection on statistics for biologists contains articles on many of the points above.

Software and code

Policy information about availability of computer code

Data collection	mass spectra were aquired with Xcalibur Version 2.2 (Thermo Scientific), UV/VIS spectra were aquired using the software that is implemented in the commercial DeNovix UV/VIS photosprectrometer, NMR data and high-resolution mass spectrometry data for detergents and detergent precursors were received from the Corefacility BioSpuramol of the Freie Universität Berlin, dynamic light scattering data were collected using the Zetasizer Software Version 7.11 (Malvern), liquid chromatography data were aquired using ChromeGate Client Viewer Version 3.3.2 (Knauer), CD spectroscopy data were aqcuired using Pro-Data Chirascan V4.5 (Applied Photophysics), fluorescence spectroscopy data were aqcuired using CLARIOstar® V5.4 (BMG Labtech)
Data analysis	mass spectra were analysed with Xcalibur version 2.2 (Thermo Scientific), ligand/lipid masses from mass spectra were calculated with Origin version 9.1 (OriginLab Corporation), mass spectra were processed with Unidec version 1.0.11, UV/VIS Data were read out manually and processed with Origin version 9.1 (OriginLab Corporation), NMR data were processed and analysed with MestReNova version 6.0.2-5475, exact masses of detergents and detergent precursors were calculated with ChemDraw version 14.0.0.117 (PerkinElmer) and compared to experimental values, dynamic light scattering data for critical aggregation concentration (cac) determination of our detergents were analysed with Origin version 9.1 (OriginLab Corporation), liquid chromatography data were analysed using ChromeGate Client Viewer Version 3.3.2 (Knauer), CD spectroscopy data were analyzed using Origin version 9.1 (OriginLab Corporation), fluorescence spectroscopy data were analyzed using MARS version 3.3 (BMG Labtech), and Origin version 9.1 (OriginLab Corporation)

For manuscripts utilizing custom algorithms or software that are central to the research but not yet described in published literature, software must be made available to editors/reviewers. We strongly encourage code deposition in a community repository (e.g. GitHub). See the Nature Research guidelines for submitting code & software for further information.

Data

Policy information about availability of data

All manuscripts must include a data availability statement. This statement should provide the following information, where applicable:

- Accession codes, unique identifiers, or web links for publicly available datasets
- A list of figures that have associated raw data
- A description of any restrictions on data availability

A list of figures and tables that have associated raw data: Figure 2b, Figure 4, Supplementary Fig. 1a, Supplementary Fig. 2a, Supplementary Fig. 3, Supplementary Fig. 4, Supplementary Fig. 5a, Supplementary Fig. 12, Supplementary Fig. 17, Supplementary Fig. 18, Supplementary Table 1 - 5

Field-specific reporting

Please select the one below that is the best fit for your research. If you are not sure, read the appropriate sections before making your selection.

[x] Life sciences [ ] Behavioural & social sciences [ ] Ecological, evolutionary & environmental sciences

For a reference copy of the document with all sections, see nature.com/documents/nr-reporting-summary-flat.pdf

Life sciences study design

All studies must disclose on these points even when the disclosure is negative.

Sample size	No sample-size calculation was performed. The applied host system was E. coli which was used for membrane protein expression. We followed previously published protein expression protocols and isolated the membranes of E. coli as stated within the Methods section of our manuscript.
Data exclusions	No data were excluded from the analysis.
Replication	We confirm that experiments were replicated and all attempts at replication were successful.
Randomization	We tested all our detergents systematically rendering randomization not necessary in our experiments.
Blinding	Our experiments did not involve subjective trials rendering blindings not necessary in our experiments.

Reporting for specific materials, systems and methods

We require information from authors about some types of materials, experimental systems and methods used in many studies. Here, indicate whether each material, system or method listed is relevant to your study. If you are not sure if a list item applies to your research, read the appropriate section before selecting a response.

Materials & experimental systems

n/a	Involved in the study	
[x]	[ ]	Antibodies
[x]	[ ]	Eukaryotic cell lines
[x]	[ ]	Palaeontology
[ ]	[x]	Animals and other organisms
[x]	[ ]	Human research participants
[x]	[ ]	Clinical data

Methods

n/a	Involved in the study	
[x]	[ ]	ChIP-seq
[x]	[ ]	Flow cytometry
[x]	[ ]	MRI-based neuroimaging

Animals and other organisms

Policy information about studies involving animals; ARRIVE guidelines recommended for reporting animal research

Laboratory animals	The study did not involve laboratory animals. The only organism involved in this study was E. coli and it was used for the overexpression of membrane proteins. E. coli membranes were isolated and extracted with our detergents.
Wild animals	The study did not involve wild animals.
Field-collected samples	The study did not involve samples field-collected samples.
Ethics oversight	No ethical approval was required because our studies did not involve laboratory animals, wild animals, or field-collected samples.

Note that full information on the approval of the study protocol must also be provided in the manuscript.
